# *In situ* hydrogel nanovaccine based on whole-cell tumor antigen efficiently activates adaptive immunity for the prevention and treatment of ovarian cancer

**DOI:** 10.1016/j.apsb.2026.07.030

**Published:** 2026-07-24

**Authors:** Jia Yan, Ruyi Lin, Qiyu Liu, Yatong Zhang, Hao Chen, Fan Tong, Yujun Song, Yulong Shi, Yuan Li

**Affiliations:** a Department of Obstetrics and Gynecology, Peking University Third Hospital, Beijing 100191, China; b Key Laboratory of Drug Targeting and Drug Delivery Systems, West China School of Pharmacy, Sichuan University, Chengdu 610041, China

**Keywords:** Ovarian cancer, Nanovaccine, Hydrogel, Whole-cell tumor antigens, CpG, Immunotherapy, Lymph node targeting, PD-1

## Abstract

Due to the late diagnosis with widespread peritoneal metastases and frequent chemoresistance, ovarian cancer has limited survival rates and necessitates innovative therapies. Here, we present an *in situ* hydrogel nanovaccine (LCMP@ALG) for spatiotemporally-synchronized antigen-adjuvant delivery to stimulate robust antitumor immunity. The hydrogel continuously releases mannosylated nanoparticles LCMP which are effectively internalized by dendritic cells and actively accumulate in lymph nodes *via* the ligand-mediated targeting effect. Benefiting from autologous tumor cell lysates, the vaccines evade antigen-specific tolerance. LCMP can synergistically activate Toll-like receptor 4 and Toll-like receptor 9 to augment antigen processing and cross-presentation, further potentiating the activation of humoral and cellular antitumor immunity. In the mouse model, the vaccine LCMP@ALG induces specific antitumor responses as well as long-term immunological memory. When combined with Programmed cell death protein 1 blockade, LCMP@ALG demonstrates a superior efficacy in prevention and elimination of ovarian cancer.

## Graphical abstract

An *in situ* hydrogel nanovaccine LCMP@ALG was constructed, and offered a multi-pronged antitumor strategy by continuously releasing nanovaccine, enhancing uptake of DCs, and simultaneously activating cellular and humoral immunity.

**Figure:**
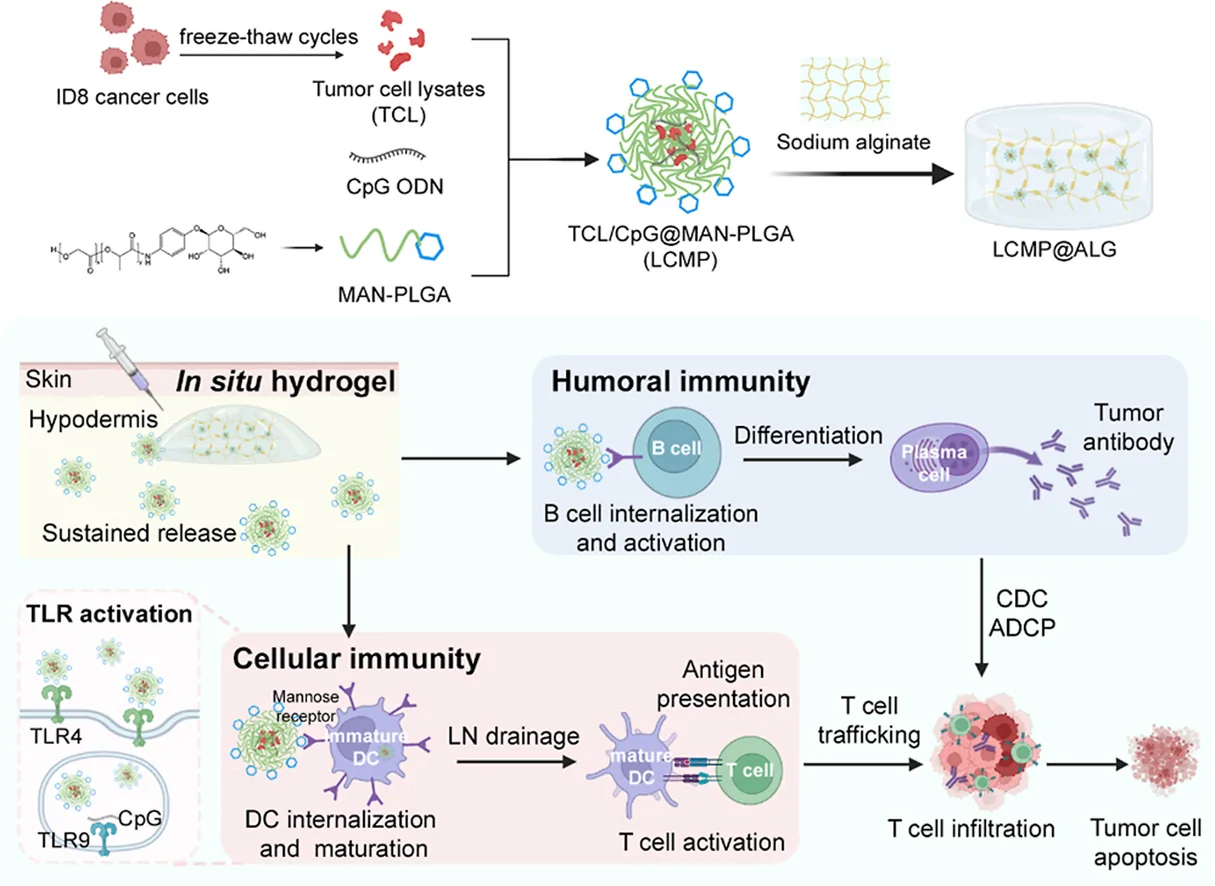

## 1 Introduction

Ovarian cancer is one of the deadliest gynecological malignancies worldwide, posing a massive threat to women's health and societal burdens^1^. The median survival of stage I ovarian cancer is up to 90% at 10 years, but <6% for stage Ⅳ^2^. Owing to the lack of effective screening and typical clinical symptoms, most patients diagnosed with ovarian cancer have advanced-stage cancer with peritoneal deposits and malignant ascites^3^^,^^4^. Therefore, effective cancer preventive strategies present a critical unmet need. Immunotherapy has become a mainstream cancer treatment in recent years^5^, ^6^, ^7^, ^8^, ^9^. Among these immunotherapies, cancer vaccines activate or restore the patient's immune system to identify and eradicate tumor cells, showing promising antitumor therapeutic and prophylactic effects^10^, ^11^, ^12^, ^13^. Substantial evidence indicates that ovarian cancer exhibits a multitude of known tumor-associated antigens, immunoreactive gene signatures, and tumor-infiltrating lymphocytes, suggesting immunotherapy could benefit ovarian cancer patients^7^^,^^14^. Thus, vaccine-based immunotherapy represents a potentially advantageous strategy for ovarian cancer.

Antigen is the core component of a powerful cancer vaccine, and using optimal tumor antigens is essential for the strength and specificity of the antitumor immune response induced by vaccines^15^, ^16^, ^17^. Tumor antigens can be classified as tumor-associated antigens (TAAs), tumor-specific antigens (TSAs), and whole-cell tumor antigens. As overexpressed self-proteins, multiple clinical trials of TAAs-based vaccines have failed due to poor immunogenicity, tumor heterogeneity and central immune tolerance^18^, ^19^, ^20^. TSAs could be exempt from immune tolerance and more recognizable by T cells, but remain challenging due to the sheer number of potential epitopes and time-consuming process of production^11^^,^^21^^,^^22^. By contrast, whole-cell tumor antigens, such as tumor cell lysates (TCL), are a simple and effective option. Due to the presence of broad-spectrum tumor antigens, whole-cell tumor antigens have been shown to overcome the shortcomings of TAAs and TSAs, while potentially eliciting more robust antitumor immune responses to significantly reduce the chance of tumor escape and recurrence^23^, ^24^, ^25^, ^26^. Moreover, the handy preparation, abundant antigen composition, and the capacity for rapid bedside generation within hours make it promising for clinical transformation into personalized vaccines^27^^,^^28^. In addition, to avoid rapid degradation or elimination of TCL *in vivo*, here we adopt poly (dl-lactide-*co*-glycolide) (PLGA), a widely used biocompatible and biodegradable polymer, to encapsulate TCL, thus achieving efficient targeting delivery and immune activation^29^.

To elicit strong antigen-specific immune responses, a potent vaccine is required to effectively target and activate antigen-presenting cells (APCs), especially dendritic cells (DCs)^30^, ^31^, ^32^. To this end, some active targeting strategies have been developed to improve the targeting ability to lymph nodes (LNs) and APCs^33^, ^34^, ^35^. The mannose receptor is highly expressed by immature DCs^36^. Mannose derivatives (MAN) modification can improve the recognition and uptake of nanoparticles by DCs, and then accumulate in LNs to enhance the antigen-specific immune response of vaccines in LNs^37^^,^^38^. Besides, MAN boosts the phenotypic maturation of DCs and B cells *via* activation of Toll-like receptor 4 (TLR4)^39^, ^40^, ^41^, ^42^. CpG oligonucleotide, a Toll-like receptor 9 (TLR9) agonist, is a well-established adjuvant that effectively improves cellular and humoral immunity^43^, ^44^, ^45^. The combination of TLR4 and TLR9 agonists has been demonstrated to polarize immune cells towards a T-helper-(Th)1 phenotype, augmenting antitumor immune responses^46^^,^^47^. Therefore, the combination of MAN and CpG is likely to elicit a more powerful adaptive immune response.

Sodium alginate (ALG) is a marketed pharmaceutical excipient capable of fast cross-linking with physiological Ca^2+^ ions upon injection^45^^,^^48^. With three-dimensional reticulate structure, hydrogels can operate as excellent drug repositories, allowing for sustained drug release and boosting therapy durability and safety^49^, ^50^, ^51^. In addition, hydrogel is suitable for subcutaneous injection, which is an ideal delivery method for tumors because of its ease of drug loading, superior biocompatibility, and biodegradability^52^, ^53^, ^54^, ^55^. Therefore, we adopt ALG to construct a nanoparticle/hydrogel dual drug release system for subcutaneous injection. Through this system, a continuous supply of tumor antigens and immune adjuvants could be provided to effectively activate immune cells^56^.

Herein, we design an *in situ* hydrogel nanovaccine, LCMP@ALG, for ovarian cancer prevention and treatment (Scheme 1). LCMP@ALG is engineered by loading TCL and CpG in the MAN modified PLGA nanoparticles to obtain a nanovaccine (LCMP) and then embedding LCMP in ALG to obtain the prefabricated hydrogel LCMP@ALG. After subcutaneous injection, LCMP@ALG cross-links with Ca^2+^ *in situ* to form a nanovaccine-loaded hydrogel, which can continuously release the nanovaccine LCMP. The MAN modification not only facilitates receptor-mediated targeting to DCs, but also activates TLR4 in the cell membrane to exert synergistic activation effect. After being internalized by DCs, LCMP simultaneously stimulates MAN-induced TLR4 and CpG-induced TLR9 signaling pathways, which contribute to the maturation and antigen-presenting capacity of DCs, thereby activating T cells to induce cellular immunity. In addition, LCMP generates humoral immunity by inducing B cells to differentiate into plasma cells, which also assists the activation of effector T cells. By continuously releasing vaccine, enhancing uptake of DCs, and simultaneously activating cellular and humoral immunity, LCMP@ALG offers a multi-pronged strategy for antitumor immune response activation, providing a novel approach for improving tumor immunotherapy.

**Scheme 1:**
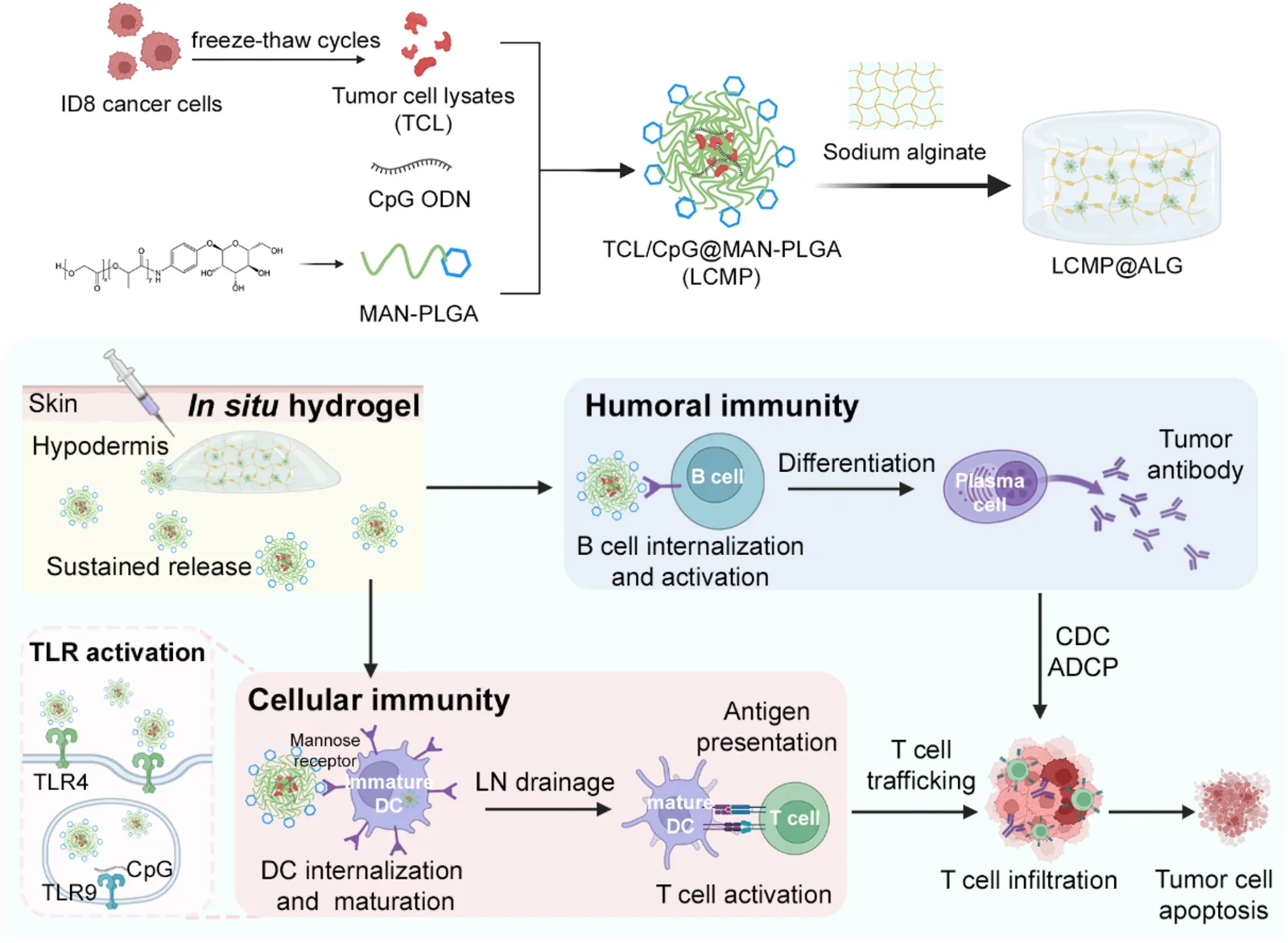
Schematic illustration of *in situ* hydrogel nanovaccine improving antitumor immune response.

## 2 Materials and methods

### 2.1 Materials

Polylactic acid-hydroxyacetic acid copolymer (PLGA, LA:GA = 50:50, Mw = 10 kDa) was purchased from Ponsure Biotechnology (Shanghai, China). CpG 1826 (5′-TCCATGACGTTCCTGACGTT-3′) was purchased from Sangon Biotech Co., Ltd. (Shanghai, China). 4-Aminophenyl-*α*-d-mannopyranoside (MAN), 4-dimethylaminopyridine (DMAP), *N*′*N*-dicyclohexyldiimide (DCC), and trimethylamine were purchased from Aladdin (Shanghai, China). Polyvinyl alcohol (PVA) and 3-(4,5-dimethylthiazol-2-yl)-2,5-diphenyltetrazolium bromide (MTT) were purchased from Biofroxx Co., Ltd. (Einhausen, Germany). Sodium alginate was purchased from Sigma–Aldrich (USA). 1,1′-Dioctadecyl-3,3,3′,3′-tetramethylindodicarbocyanine, 4-chlorobenzenesulfonate salt (DiD) and Coumarin 6 (Cou-6) were purchased from J&K Scientific Technology Co., Ltd. (Beijing, China). Enzyme-linked immunosorbent assay (ELISA) kits were purchased from Thermo Fisher Scientific Inc. (USA). Lipopolysaccharide (LPS) was obtained from Invitrogen (USA). All the antibodies for flow cytometry were purchased from BioLegend (USA). Anti-PD-1 antibody was purchased from BioXCell (USA).

### 2.2 Cell line and animals

Murine ID8 ovarian cancer cells, ID8-Luc cells, and DC2.4 cells were obtained from the Chinese Academy of Sciences Cells Bank (Shanghai, China). Bone marrow-derived dendritic cells (BMDCs) were isolated from mouse bone marrow in femurs and tibias. The cells were cultured in complete RPMI 1640 or Dulbecco's modified Eagle's medium, consisting of 10% fetal bovine serum, penicillin G (100 U/mL), and streptomycin sulfate (100 U/mL).

Female C57BL/6 mice (5–6 weeks, 18–20 g) were purchased from SiPeiFu Biotechnology Co., Ltd. (Beijing, China). All animal experiments were performed under the guidelines and evaluated and approved by the ethics committee of Sichuan University (K2022011).

### 2.3 Synthesis of MAN-PLGA

MAN-PLGA was performed as previously described^47^, with minor modifications (Supporting information Scheme S1). Briefly, PLGA (0.0125 mmol) and DCC (0.025 mmol) were dissolved in *N*,*N*-dimethylformamide and activated by stirring at room temperature for 15 min. DMAP (0.05 mmol) was added to the previous solution and stirred for 5 min at room temperature. MAN (0.025 mmol) and 10 μL triethylamine were added and stirred at 35 °C for 48 h under a nitrogen atmosphere. Deionized water was used to precipitate the polymer, which was then recovered using centrifugation. The polymer was dissolved in dichloromethane and dried using anhydrous sodium sulfate. The supernatant was then concentrated by rotary evaporation, precipitated with diethyl ether, and dried in desiccators under vacuum overnight. The products were characterized by proton nuclear magnetic resonance (^1^H NMR) spectroscopy (Agilent Tech UNITY INOVA 400 spectrometer), Fourier transform infrared (FTIR) spectroscopy (Bruker, INVENIOR), and Ultraviolet–visible (UV–Vis) spectroscopy (Thermo Fisher Scientific, GENESYS 180).

### 2.4 Preparation and characterization of nanoparticles

The double emulsion approach was used to prepare LCMP as follows: 50 μg of CpG was dissolved in 100 μL of 5 mg/mL TCL as the water phase. 10 mg MAN-PLGA was dissolved in 1 mL acetone-dichloromethane (1:1, *v*/*v*) as the organic phase. Then the water phase was added to the organic phase in an ice bath and sonicated (150 W, 5 s/5 s a cycle for 2 min). The primary emulsion was added to 5 mL of 2% PVA solution and sonicated in an ice bath for another 2 min using the same parameters. The double emulsion was evaporated under reduced pressure at 30 °C to remove organic solvent. Next, particle suspension was centrifuged (2000×*g*, 10 min, 4 °C) to remove the nonencapsulated payloads and washed in ultrapure water three times (15,000×*g*, 30 min, 4 °C) before suspension. TCL@PEG-PLGA (LPP), TCL@MAN-PLGA (LMP) and TCL/CpG@PEG-PLGA (LCPP) were prepared similarly.

The particle size, distribution, and *zeta* potential of these nanoparticles were measured by laser sizer (Brookhaven, USA). Transmission electron microscopy (TEM) (H-600, Hitachi, Japan) was used to determine the morphology of these nanoparticles. Sodium dodecyl sulfate-polyacrylamide gel electrophoresis (SDS-PAGE) and Coomassie brilliant blue (Beyotime, P0017A) were employed to determine the protein profiles of LCMP.

To quantify encapsulated TCL and CpG, nanoparticles were dissolved in 0.1 mol/L NaOH and 0.1% SDS at 37 °C overnight, then the BCA protein assay kit (Beyotime, P0010) was used to quantify the amount of TCL, and fluorescence spectroscopy (VICTOR Nivo, PerkinElmer, USA) was used to quantify the amount of FAM-CpG. To further evaluate the stability, nanoparticles were incubated under conditions of 100 rpm at 4 or 37 °C, and particle size and polymer dispersity index (PDI) were measured by laser sizer.

### 2.5 Preparation and characterization of hydrogel

Prefabricated hydrogels were prepared by mixing nanoparticles with ALG solution (final ALG concentration = 10 mg/mL). Free TCL and CpG with a mass ratio of 1:0.15 were mixed with ALG to obtain (TCL + CpG)@ALG. *In vitro*, this prefabricated hydrogel was exposed to CaCl_2_ solution to form hydrogels. Scanning electron microscopy (SEM) (SU3500, Hitachi, Japan) was used to observe the structure of hydrogels. Rheological instrumentation (MCR302, Anton Paar, Austria) was used to evaluate the rheological properties of hydrogels.

The equilibrium swelling ratio (SR) of gels was evaluated by gravimetric method. Briefly, a certain weight (m_0_) of the gel was incubated in PBS at 37 °C, and gel mass (m_*t*_) was examined after removing free water at predetermined time points. The SR of the gels was calculated by Eq. (1):

(1) $$\text{SR}=\frac{m_{t}-m_{0}}{m_{0}}\times 100\%$$

To evaluate drug release, 100 μL LCMP@Gel was immersed in 1 mL PBS under conditions of 80 rpm at 37 °C. At preset time points, 0.2 mL of release medium samples were removed and replaced with an equal volume of fresh PBS. TCL concentrations were examined by BeyoBCA protein assay kit (Beyotime, P0399S), and CpG concentrations were examined by fluorescence spectroscopy.

### 2.6 Cell viability *in vitro*

Biocompatibility of different formulations on DCs was investigated by MTT assay. DC2.4 cells (6 × 10^3^ per well) were placed into 96-well plates and grown for 24 h. The cells were stimulated with different concentrations of TCL + CpG, MP, and LCMP, while fresh medium was used as a control. 24 h later, MTT (10 μL, 5 mg/mL) was added for 4 h. Then 100 μL DMSO solution replaced the culture medium, and the cell viability was measured at 570 nm by using a microplate reader.

### 2.7 Cellular uptake *in vitro*

For quantitative analysis, BMDCs and DC2.4 cells were planted in 24-well plates and grown to reach 80%. The cells were incubated with Cou-6-loaded nanoparticles for 1, 4, and 8 h. Then, cells were collected and analyzed by flow cytometry (Agilent, NovoCyte). For qualitative analysis, DC2.4 cells were placed in 24-well plates pre-coated with coverslips and treated as described above. Next, the cells were washed, fixed, stained with 4′,6-diamidino-2-phenylindole (1 μg/mL), and observed by confocal microscopy (Leica, DM2000 LED).

### 2.8 DCs maturation *in vitro*

To optimize the ratio of TCL and CpG, BMDCs (5 × 10^5^ per well) were planted into 12-well plates and incubated with LMP (at the same concentration of 2 μg TCL) and different concentrations of CpG for 24 h. The BMDCs were washed, stained with anti-CD11c-FITC, anti-CD80-PE/Cy7, and anti-CD86-PE antibodies, and analyzed by flow cytometry.

To evaluate the immunological stimulation induced by LCMP, BMDCs (5 × 10^5^ per well) were planted into 12-well plates and incubated with different formulations (at the same concentration of 2 μg TCL, 1 μg/mL LPS as positive control) and administered as described above. The levels of interleukin (IL)-1*β*, IL-12 and tumor necrosis factor-*α* (TNF-*α*) in medium supernatants were detected using ELISA kits according to the manufacturer's instructions.

### 2.9 Activation of T cells and B cells *in vitro*

To explore the proliferation of CD8^+^ T cells by BMDCs treated with different formulations, BMDCs (3 × 10^5^ per well) were planted into 12-well plates and incubated with different formulations for 24 h. Then BMDCs were co-incubated with CFSE-labeled lymphocytes (1 × 10^6^ per well) isolated from mouse spleens for 72 h. The cells were washed, stained with anti-CD3-PE/Cy7 and anti-CD8-APC antibodies, and analyzed by flow cytometry. The levels of IL-6 and IL-12 in cell supernatant were detected using ELISA kits.

To explore the differentiation of B cells by different formulations, lymphocytes (1 × 10^6^ per well) were planted into 12-well plates and incubated with different formulations for 48 h. The cells were washed, stained with anti-CD19-APC, anti-CD86-PE, and anti-CD138-PE/Cy7 antibodies, and analyzed by flow cytometry. The levels of Interferon-*γ* (IFN-*γ*), IL-12, and TNF-*α* in cell supernatant were detected using ELISA kits.

### 2.10 Specific killing effect on tumor cells *in vitro*

To explore the specific killing effect on ID8 tumor cells by activated T cells and B cells, BMDCs (3 × 10^5^ per well) were planted into 12-well plates and incubated with different formulations for 24 h. Then BMDCs were co-incubated with lymphocytes (1 × 10^6^ per well) isolated from mouse spleens for 48 h. The ID8 cells were seeded in 96-well plates (5 × 10^3^ cells per well) for 12 h. Next, activated lymphocytes in different groups were co-incubated with ID8 cells in 96-well plates for 48 h. The killing effects of immune cells could be evaluated by measuring lactate dehydrogenase (LDH) activity in tumor cells. LDH activity was examined by LDH Cytotoxicity Assay Kit with WST-8 (Beyotime, C0018S) according to the manufacturer's protocol.

### 2.11 Biodistribution *in vivo*

DiD-labeled PP@ALG and MP@ALG (0.25 mg/kg for DiD) were subcutaneously injected into female C57BL/6 mice at the lower dorsal region (near the tail base). For semiquantitative research, after administration for 1 day or 7 days, LNs and major organs (heart, liver, spleen, lungs, and kidneys) were collected and subjected to *ex vivo* fluorescence images by the *in vivo* imaging system (IVIS) imaging system (LIVIS Lumina III, PerkinElmer, USA). Then LNs and major organs were fixed, dehydrated, frozenly sectioned, stained with DAPI, and imaged by confocal microscopy. For quantitative research, after 1 day, draining LN (dLNs) were collected, dispersed into single-cell suspensions, and analyzed by flow cytometry.

### 2.12 Immune response after a single vaccination

Female C57BL/6 mice were subcutaneously injected with different formulations, including PBS (as control), (TCL + CpG)@ALG, LPP@ALG, LCPP@ALG, and LCMP@ALG (1.25 mg/kg for TCL per mouse). After 48 h, the LNs and spleens were collected, dispersed into single-cell suspensions, and analyzed by flow cytometry. To label mature DCs, splenocytes and lymphocytes were stained with CD11c, CD80, CD86, and major histocompatibility complex (MHC) I (H-2K^b^). Subsequently, splenocytes were stained with CD19, CD86, and CD138 to investigate activated B cells and plasma cells. CD3, CD8, CD4, and CD69 were applied for active T cells. In addition, splenocytes were co-stained with CD3, CD8, and CD25 to detect the levels of active CD8^+^ T cells in the spleen. Furthermore, the proportion of IFN-*γ*-secreting cytotoxic T lymphocytes involved the application of CD3, CD8, and IFN-*γ*.

### 2.13 Antitumor efficacy *in vivo*

For tumor prophylactic experiments, female C57BL/6 mice were randomly divided into five groups and subcutaneously injected with different formulations, including PBS (as control), (TCL + CpG)@ALG, LPP@ALG, LCPP@ALG, and LCMP@ALG (1.25 mg/kg for TCL per mouse), weekly for 3 times. The mice were injected with 2 × 10^6^ ID8-luc cells 2 days post last vaccination. Tumor burden was monitored using the IVIS imaging system. The body weight and survival rates were recorded. Mice in each group were sacrificed 16 days after the injection of tumor cells. Major organs, uterine appendages, and intestines were collected and subjected to obtain *ex vivo* fluorescence images.

For tumor therapeutic experiments, C57BL/6 mice were injected with 1 × 10^7^ ID8-luc cells. After 7 days, these tumor-bearing mice were randomly divided into six groups and subcutaneously injected with different formulations, including PBS (as control), (TCL + CpG)@ALG, LPP@ALG, LCPP@ALG, LCMP@ALG, and LCMP@ALG + anti-PD-1 (1.25 mg/kg for TCL per mouse), weekly for 4 times. The PD-1 antibody (100 μg per mouse) was injected intraperitoneally in the LCMP@ALG + anti-PD-1 group 24 h after LCMP@ALG administration. Tumor burden was monitored using the IVIS imaging system. Mice in each group were sacrificed 45 days after the injection of tumor cells. LNs, spleens, and ascites were collected to evaluate the immune response. Meanwhile, blood samples were collected for blood biochemical tests. Major organs and skin at the site of administration were collected and used for Hematoxylin & Eosin (H&E) staining.

### 2.14 Antitumor immune response *in vivo*

The isolated LNs, spleens, and ascites were dispersed into single-cell suspensions through mechanical grinding and filtering. Flow cytometry was used to explore the antitumor immunity effects. The DCs maturation and activation of T cells and B cells were investigated as mentioned above. Furthermore, splenocytes were also stained with anti-CD3-PE/Cy7, anti-CD8-APC, anti-CD4-APC/Cy7, anti-CD44-FITC, and anti-CD62L-PE to evaluate the level of central memory T cells (Tcm). In addition, CD3-PE/Cy7, anti-CD4-FITC, and anti-Foxp3-PE antibodies were used to stain ascites to detect regulatory T cells (Tregs).

### 2.15 Statistical analysis

GraphPad Prism software 10.0 was applied for statistical analysis. Data were presented as mean ± standard deviation (SD). All the statistical analysis was assessed using one-way analysis of variance (ANOVA) for comparison among multiple groups, two-way ANOVA for comparison among multiple groups with different time points, or *t* test for multiple comparisons. Statistical significance was set at the following levels: ∗*P* < 0.05, ∗∗*P* < 0.01, ∗∗∗*P* < 0.001, ∗∗∗∗*P* < 0.0001, and “ns” stands for no significant difference.

## 3 Results and discussion

### 3.1 Preparation and characterization of LCMP@ALG

The synthesis of the MAN-grafted PLGA polymer was confirmed by ^1^H NMR, FTIR, and UV spectra. As shown in Fig. 1A, the chemical shifts of MAN (*δ* = 6.84, 6.49) and the peak of the amide bond (*δ* = 8.41, 8.24) in the ^1^H NMR spectra clearly indicated the successful modification of MAN on PLGA. The characteristic absorption bands at 1670 and 1510 cm^−1^ in the FTIR spectrum (Supporting information Fig. S1), and characteristic MAN absorption peak at 300 nm in the UV spectrum (Fig. 1B), further confirmed the successful modification of PLGA with MAN. To further optimize the ratio of TCL and CpG, the activation effect on APCs of different mass ratios of TCL and CpG was evaluated on BMDCs maturation *in vitro*. Compared with other ratios, the 1:0.15 ratio of TCL to CpG induced the highest percentages of the co-stimulatory molecules CD80 and CD86 in BMDCs (Fig. 1C). Therefore, the mass ratio of 1:0.15 for TCL and CpG was selected in subsequent experiments investigating antitumor immune responses. A water-in-oil-in-water (w/o/w) emulsion method was used to fabricate nanoparticles. After screening, the ratio of TCL and CpG encapsulated in the nanoparticles was close to 1:0.15, where the dosages of TCL and CpG were 0.5 mg and 75 μg, respectively (Supporting information Tables S1 and S2). LCMP presented a negative surface charge (−17.6 ± 0.5 mV) and relatively small hydrodynamic diameter (159.6 ± 0.9 nm) with narrow distribution (PDI = 0.145 ± 0.030), which were similar to LPP and LCPP (Fig. 1D and E, Supporting information Fig. S2). TEM revealed that LCMP presented uniform spherical morphology with an outer shell and an inner core (Fig. 1F). LCMP lysates were separated by SDS-PAGE to compare the protein patterns in LCMP. As shown in Fig. 1G, the protein patterns of LCMP were similar to those of TCL, suggesting an extensive retention of proteins in LCMP. After incubation with LCMP, Concanavalin A (Conc A) formed aggregates with exposed mannose residues, revealed by an increase in average size and a time-dependent increase in the absorbance indices (Supporting information Fig. S3), indicating the MAN was displayed on the surface of the LCMP. Besides, colloidal stability is a key indicator for wide use of nanoparticles. During the 10-day incubation, the hydrodynamic size and PDI of LCMP did not change significantly, indicating its good stability (Fig. 1H, Supporting information Fig. S4).

**Figure 1:**
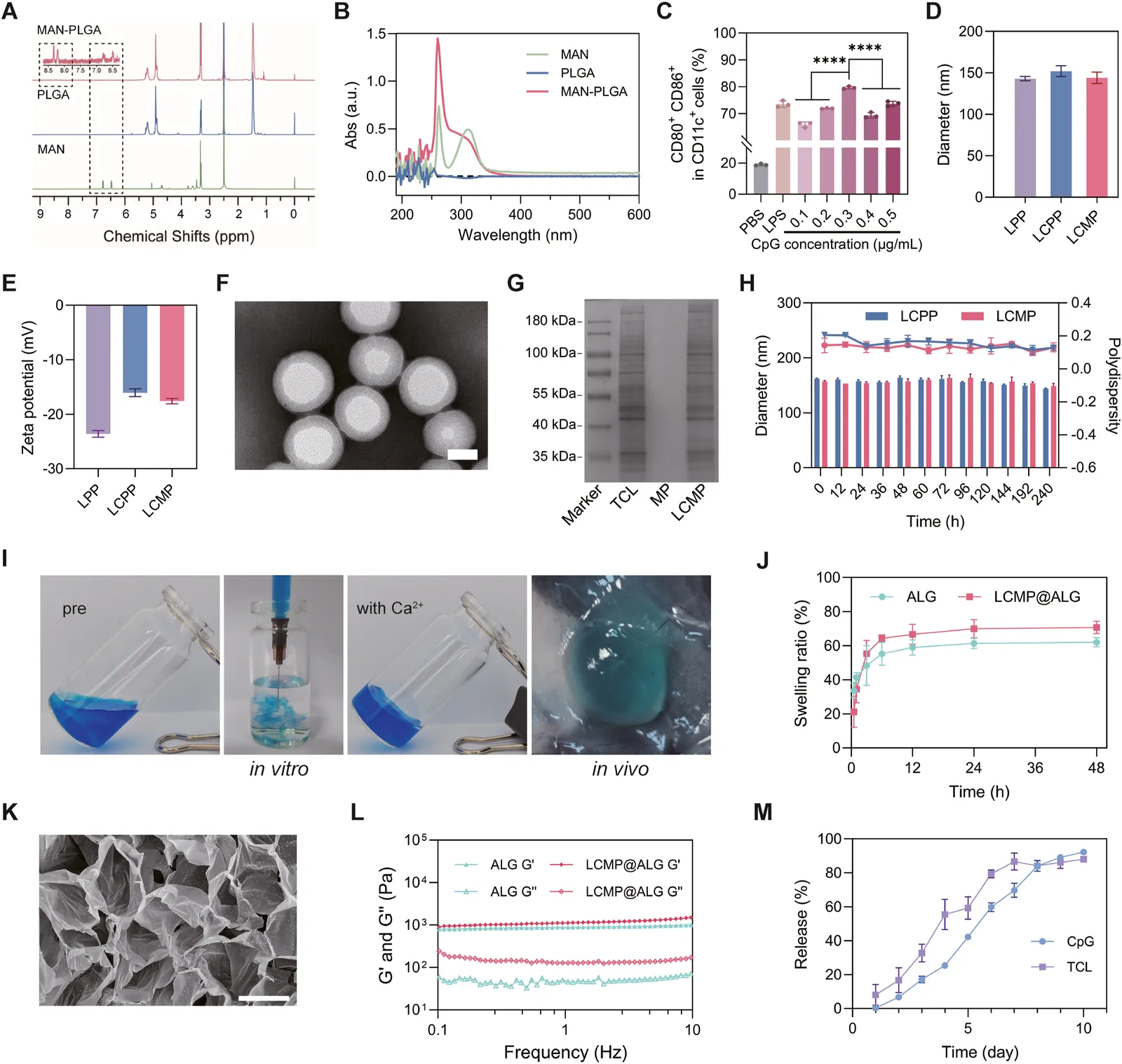
Characterization of LCMP@ALG. (A) ^1^H NMR spectrum of MAN, PLGA, and MAN-PLGA. (B) UV absorption spectra of MAN, PLGA, and MAN-PLGA. (C) Flow cytometry analysis of CD80^+^CD86^+^ cells in BMDCs treated with different ratios of TCL and CpG after 24 h. The (D) hydrodynamic diameter and (E) *zeta* potential of LPP, LCPP, and LCMP. (F) TEM image of LCMP. Scale bar = 50 nm. (G) SDS-PAGE protein analysis of TCL, MP, and LCMP. (H) Stability of LCPP and LCMP for 24 h. (I) Photographs showing the formation of hydrogels *in vitro* and *in vivo*. (J) Swelling ratio of hydrogel. (K) SEM image of LCMP@ALG. Scale bar = 100 μm. (L) Frequency sweep tests of hydrogels. (M) Cumulative release profiles of TCL and CpG. Data are presented as mean ± SD (*n* = 3). ∗∗∗∗*P* < 0.0001, and “ns” stands for no significant difference.

To construct the sustained release system, LCMP was further mixed with ALG solution to form the prefabricated hydrogel LCMP@ALG. When exposed to a Ca^2+^ solution, this prefabricated hydrogel demonstrated the potential for forming *in situ* hydrogels. To determine the hydrogel formation capacity of ALG, a 1 mL syringe was used for injection and it was discovered that the prefabricated hydrogel had good injectable characteristics, generating a homogeneous hydrogel in the Ca^2+^ solution (Fig. 1I). SEM imaging provided further insight into the hydrogel scaffolds' structural arrangement, showing an interconnected porous loose network structure (Fig. 1K, Supporting information Fig. S5). Moreover, LCMP@ALG demonstrated rapid swelling by 6 h and reached equilibrium at about 24 h (Fig. 1J). The LCMP@ALG's mechanical strength and toughness were examined using rheological measures. From the stress sweep tests and frequency sweep tests, the storage moduli (G′, measurement of elastic properties) remained higher than the loss moduli (G″, measurement of viscous properties), indicating the formation of viscoelastic solid-like hydrogel (Supporting information Fig. S6A and S6B). Notably, G′ of LCMP@ALG was slightly higher than that of blank ALG hydrogel, indicating that the introduction of LCMP in the ALG hydrogel could improve its mechanical properties (Fig. 1L). The viscosity measurement exhibited shear-thinning property of LCMP@ALG (Fig. S6C). Furthermore, TCL and CpG could be released continuously from ALG hydrogel, with total release taking roughly 7–10 days (Fig. 1M), implying that the ALG hydrogel had a long-term sustained release effect, offering the benefit of a long-acting vaccine.

### 3.2 Characterization of LCMP *in vitro*

Since the key role of APCs in cancer vaccination has been proven, it was investigated whether the integrated nanovaccine, LCMP, could significantly stimulate BMDCs *in vitro*. For safety confirmation, the cytotoxicity of formulations was evaluated using MTT assay. After incubating with DC2.4 cells for 24 h, no obvious cytotoxicity was observed in all experimental groups at all concentrations (Fig. 2A), demonstrating the suitable physiological biocompatibility of LCMP. Given that internalization into APCs is the starting phase of antigen processing and maturation, Cou-6-loaded nanoparticles were prepared to examine the uptake of the different formulations by DCs. Nanoparticles modified with MAN (MP) exhibited higher fluorescence intensity in DC cells compared with nanoparticles modified with PEG (PP) due to the overexpression of MAN receptors on DCs (Fig. 2B–D).

**Figure 2:**
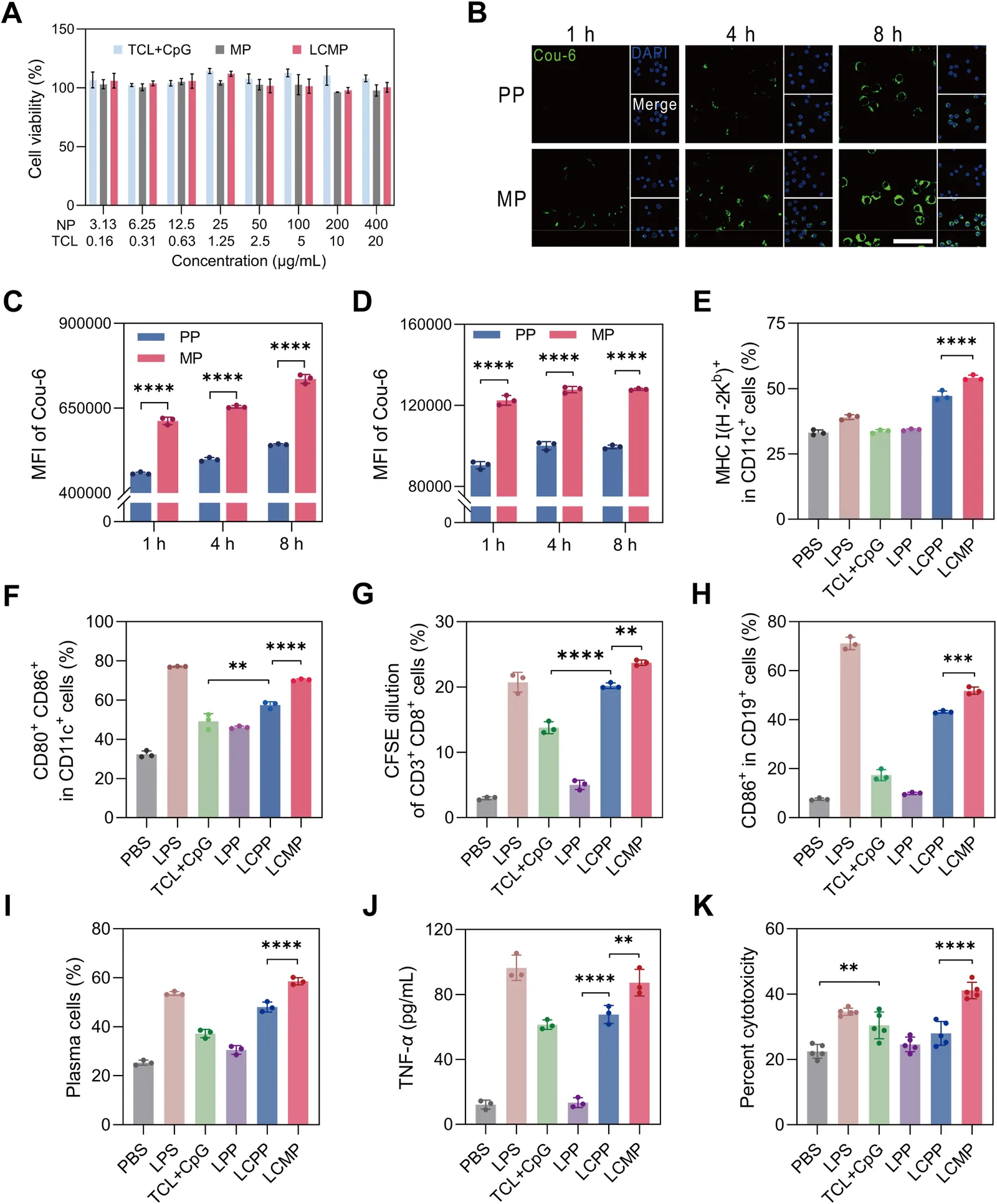
Evaluation of the activation of immune cells *in vitro*. (A) The cell viability of different formulations was assessed by MTT assay (*n* = 3). Cellular uptake analysis of Cou-6@PP and Cou-6@MP for 1, 4, and 8 h in DC2.4 cells evaluated by (B) confocal microscopy and (C) flow cytometry. Scale bar = 50 μm. (D) Cellular uptake analysis of Cou-6@PP and Cou-6@MP for 1, 4, and 8 h in BMDCs evaluated by flow cytometry. Flow cytometry analysis of (E) MHC Ⅰ^+^ cells and (F) CD80^+^CD86^+^ cells in BMDCs treated with different formulations for 24 h. (G) Flow cytometry analysis of CFSE dilution in CD8^+^ T cells after co-culture with BMDCs for 72 h. Flow cytometry analysis of (H) CD86^+^ B cells and (I) plasma cells in lymphocytes treated with different formulations for 48 h. (J) TNF-*α* release from lymphocytes treated with different formulations for 48 h. (K) Percentage of cytotoxic activity evaluated by LDH released. Data are presented as mean ± SD. ∗∗*P* < 0.01, ∗∗∗*P* < 0.001, ∗∗∗∗*P* < 0.0001, and “ns” stands for no significant difference.

The maturation of DCs is imperative for activating antitumor immune responses. The BMDCs, treated with the different formulations for 24 h, were evaluated for the expression of co-stimulatory molecules and MHC I. As expected, compared to LPP group, the levels of co-stimulation molecules CD80 and CD86 in the LCPP group exhibited significant changes, implying that the co-delivery of antigen and adjuvant is essential for effective immune activation (Fig. 2F). By contrast, BMDCs incubated with LCMP exhibited significantly higher levels of CD80^+^CD86^+^ DCs than those incubated with LCPP, mainly due to the enhanced uptake and activation of TLR4 by MAN modification. As shown in Supporting information Fig. S7, the TLR4 inhibitor, schaftoside, significantly inhibited BMDCs maturation, confirming the involvement of TLR4 in this process. These results collectively demonstrate that simultaneous TLR4 and TLR9 activation synergistically promotes DCs maturation. In addition, the same trend was also exhibited in the expression of MHC I (Fig. 2E), suggesting the higher antigen cross-presenting ability of BMDCs treated with LCMP. Since the APC-derived cytokines are necessary for the recruitment and activation of immune cells, pro-inflammatory cytokines released by BMDCs were also examined. As shown in Supporting information Fig. S8, BMDCs treated with LCMP exhibited highest levels of IL-1*β*, IL-12, and TNF-*α* when compared to those treated with other formulations. Therefore, LCMP markedly enhanced DCs maturation and antigen presentation, along with high expression of soluble immunostimulatory molecules, all critical for generating effective adaptive immunity.

Next, the capacity to promote T cell immunity was assessed *in vitro*. In contrast to BMDCs treated with other formulations, BMDCs treated with LCMP considerably promoted CD8^+^ T cell proliferation, almost 8-fold more than the PBS group (Fig. 2G), and stimulated higher IL-6 and IL-12 release (Supporting information Fig. S9), which was a clear sign of the activation of antigen-specific CD8^+^ T cells. These results demonstrate that LCMP can effectively improve BMDCs maturation through dual activation of TLR4 and TLR9 and innate immunity stimulation, significantly boosting T cell proliferation *via* LCMP-treated BMDCs.

The *in vitro* stimulation effects of LCMP on B cells were verified by evaluating the phenotypes of the cells and the levels of secreted cytokines. TCL and CpG both activated B cells; however, LPP just slightly improved the percentage of activated B cells, which was significantly lower than that in LCPP group (Fig. 2H), suggesting CpG played the major role in activating B cells. The percentage of activated B cells in the LCMP group was significantly higher than that in the LCPP group owing to the simultaneous activation of dual signal pathways. Similarly, the percentage of plasma cells was also upregulated by LCMP (Fig. 2I), indicating that LCMP could enhance both the antigen-presenting and antibody-secreting capacity of B cells. Furthermore, markedly elevated concentrations of IFN-*γ*, IL-12 and TNF-*α* in LCMP group were consistent with the trend of plasma cells (Fig. 2J, Supporting information Fig. S10).

Next, it was investigated whether activated lymphocytes, induced by LCMP-pulsed BMDCs, could demonstrate the particular killing capacity towards tumor cells *in vitro*. ID8 cells were co-cultured with lymphocytes activated by different formulations-pulsed BMDCs for 48 h. LDH is an extensively used biomarker for cell death. As so, the LDH assay was carried out to investigate LDH release from ID8 cells and discovered that only activated T cells in the LCMP group significantly enhanced LDH release from ID8 cells (Fig. 2K). These data suggested that LCMP could activate both cellular immunity and humoral immunity simultaneously to generate tumor-specific killing effects *in vitro*, indicating the possibility of targeted tumor prevention *in vivo*.

### 3.3 Distribution of LCMP and its induced immune responses *in vivo*

Since LNs are known to be crucial for antitumor immune responses, nanovaccines should stimulate T cells activation in dLNs. Decorating drug delivery systems with ligands targeting APCs facilitates the accumulation of drugs in LNs, which can improve antitumor efficacy and reduce toxic side effects. To further investigate the LNs targeting ability of LCMP, the accumulation of LCMP in dLNs was assessed by using IVIS. DiD-loading PP@ALG and MP@ALG were injected subcutaneously into C57BL6N mice. At predetermined time points, LNs were harvested to obtain *ex vivo* fluorescence imaging. The fluorescence intensity of dLNs in DiD@MP group was 1.85- and 1.59-fold higher than that of DiD@PP group, respectively (Fig. 3A and B). Then, the DiD fluorescence in DCs was also analyzed by flow cytometry at 1 day post injection. The uptake of DiD@MP by DCs within dLNs was stronger than that of DiD@PP (Fig. 3E), which was similar to the results of cellular uptake *in vitro*. Immunofluorescence imaging further demonstrated that LNs resected from the DiD@MP groups were more accumulated than those from the DiD@PP group (Fig. 3D), demonstrating that DiD@MP can deeply infiltrate into LNs. In addition, the distribution of DiD@MP in main organs was investigated. As shown in Fig. 3C and Supporting information Figs. S11 and S12, nanoparticles were mainly distributed in the liver and spleen, and the fluorescence intensity in the spleen of DiD@MP was higher than that of DiD@PP, which resulted from the migration of immune cells that internalized DiD@MP. These results validated that MAN coating facilitates lymphatic drainage.

**Figure 3:**
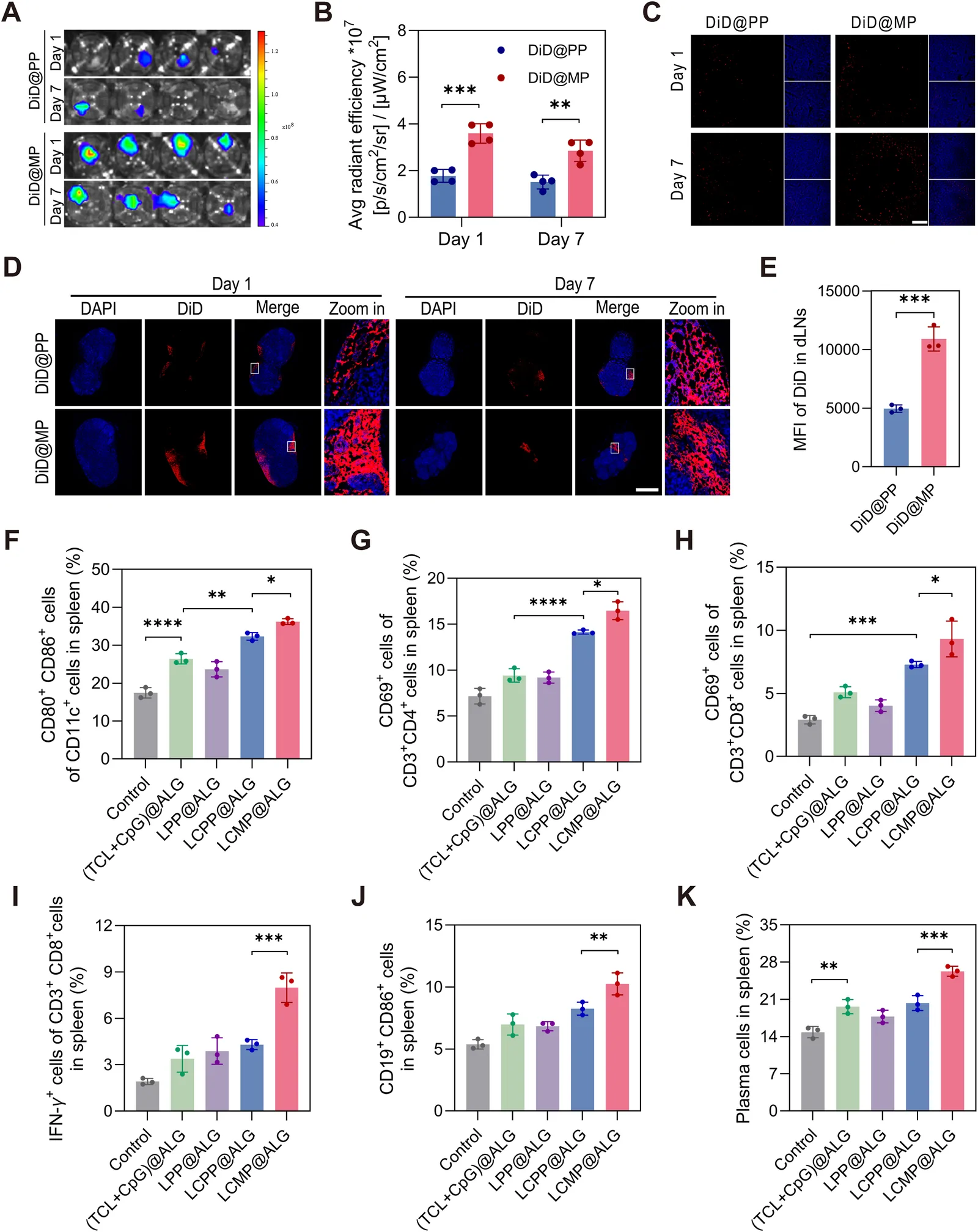
*In vivo* distribution and stimulation of systemic immunity. (A) IVIS images and (B) semiquantitative data of dLNs (*n* = 4). (C) Fluorescent images of frozen sections of spleens. Scale bar = 100 μm. (D) Fluorescent images of frozen sections of dLNs. Scale bar = 1 mm. (E) Flow cytometry analysis of intracellular fluorescence intensity of DCs in dLNs (*n* = 3). (F) Flow cytometry analysis of CD80^+^CD86^+^ cells in the spleen *in vivo* 48 h after treatment (*n* = 3). The percentages of CD69^+^ cells in (G) CD4^+^ T cells and (H) CD8^+^ T cells in the spleen *in vivo* evaluated by flow cytometry (*n* = 3). Flow cytometry analysis of (I) IFN-*γ*^+^ CD8^+^ T cells in the spleen *in vivo* 48 h after treatment (*n* = 3). Flow cytometry analysis of (J) CD19^+^CD86^+^ cells and (K) plasma cells in the spleen *in vivo* 48 h after administration (*n* = 3). Data are presented as mean ± SD. ∗*P* < 0.05, ∗∗*P* < 0.01, ∗∗∗*P* < 0.001, ∗∗∗∗*P* < 0.0001, and “ns” stands for no significant difference.

Subsequently, we investigated the potential immunological responses generated by LCMP@ALG *in vivo* after a single immunization. Firstly, the co-stimulatory signals on DCs in the dLNs and spleens were evaluated. As expected, MHC I expression and the co-stimulatory markers CD80 and CD86 on DCs increased significantly in the LCMP@ALG groups (Fig. 3F, Supporting information Fig. S13). Further, the T cell activation in the spleen was investigated. There was the highest frequency of CD69^+^CD4^+^ T cells and CD69^+^CD8^+^ T cells in the spleen of LCMP treated mice, whose percentages of CD69^+^CD4^+^ T cells and CD69^+^CD8^+^ T cells were 2.30- and 3.20-fold higher than those in control groups, respectively (Fig. 3G and H, Supporting information Fig. S14). Similarly, there were higher percentages of activated CD8^+^ T cells (CD25^+^CD8^+^ T cells and IFN-*γ*^+^CD8^+^ T cells) in the spleen of LCMP@ALG treated mice than in other groups (Fig. 3I, Supporting information Fig. S15). Meanwhile, the effect of various formulations on B cells was evaluated. The LCMP@ALG group exhibited a 1.91-fold higher percentage of activated B cells and a 1.77-fold higher percentage of plasma cells than that in the control group, mainly due to dual activation of TLR4 and TLR9 (Fig. 3J and K, Supporting information Fig. S16). Moreover, the LCMP@ALG group also observed distinctly elevated concentrations of IL-1*β* and IFN-*γ* in the spleen, indicating that systemic immune responses were initiated (Supporting information Fig. S17). Collectively, these data demonstrate that LCMP@ALG can effectively induce antitumor immunity by activating DCs and draining into dLNs, which is compatible with our *in vitro* results.

### 3.4 Antitumor effect *in vivo*

Motivated by the results that LCMP@ALG can reliably elicit both cellular and humoral immune responses, we next investigated the possible preventative value of immunization with LCMP@ALG in the context of tumor growth. Briefly, C57BL/6 mice were randomized into five groups and immunized with different formulations weekly for 3 weeks. Then ID8 cells were injected intraperitoneally into each C57BL/6 mouse (Fig. 4A). IVIS Spectrum imaging was used to assess tumor burden *via* bioluminescence. As shown in Fig. 4B and D, owing to the CpG-mediated immunomodulation, (TCL + CpG)@ALG and LCPP@ALG delayed the induction and growth of ovarian cancer, whose effect was better than LPP@ALG. And LCMP@ALG treated mice displayed more significant tumor suppression than other groups. This result was further corroborated by the bioluminescence signals in *ex vivo* major organs: a considerable decrease of ID8-Luc tumor nodules in the intestine was discovered after LCMP@ALG treatment (Fig. 4E). As the survival curve shown (Fig. 4C), LCMP@ALG led to the best protection against ovarian cancer challenge with the longest median survival (66 days), which was 1.27-fold longer than mice in control groups. This result was further confirmed by individual body circumference curves (Fig. 4F–J).

**Figure 4:**
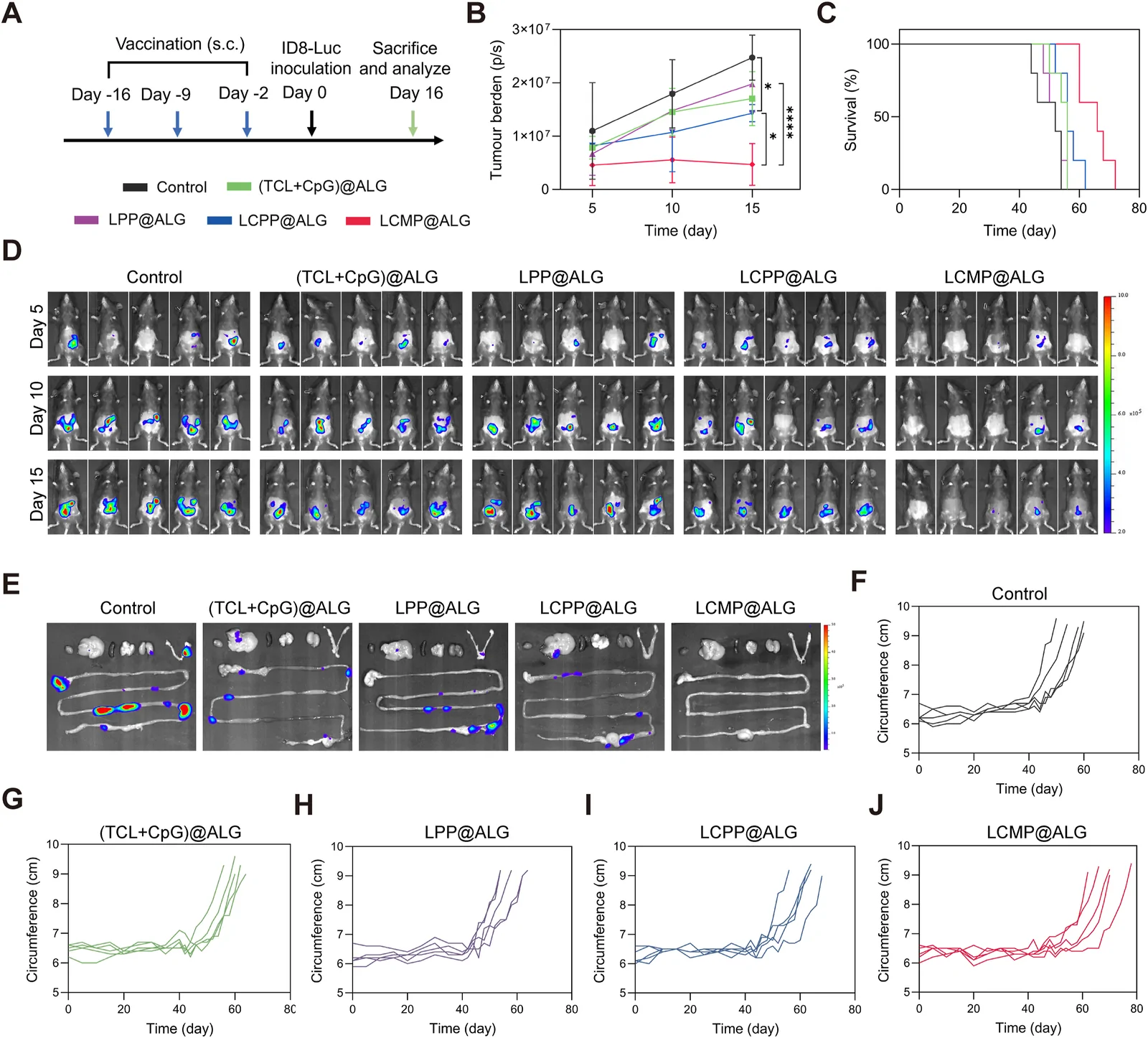
Prevention of tumor progression by LCMP@ALG. (A) Treatment timeline for preventing tumors. (B) Semi-quantification data, (C) survival curves, and (D) *in vivo* bioluminescence imaging of ID8-Luc tumor-bearing mice (*n* = 5). (E) The *ex vivo* bioluminescence imaging of major organs. Individual circumferences in (F) control group, (G) (TCL + CpG)@ALG group, (H) LPP@ALG group, (I) LCPP@ALG group, and (J) LCMP@ALG group (*n* = 5). Data are presented as mean ± SD. ∗*P* < 0.05, ∗∗∗∗*P* < 0.0001, and “ns” stands for no significant difference.

In addition to the preventive effect of ovarian cancer, the effectiveness of LCMP@ALG on cancer therapy was also validated. Additionally, PD-1 blockade was further employed to improve antitumor immunity. As shown in Fig. 5A, the tumor-bearing mice received four doses of respective formulations on Days 7, 14, 21, and 28, and an additional four doses of anti-PD-1 on Days 8, 15, 22, and 29. On Day 45, the mice were sacrificed, and the major organs and ascites were collected for further investigation. Due to the direct injection of ID8 cells into the peritoneal cavity, these cells resulted in significant tumor deposits on the peritoneal wall and peritoneal organs, along with hemorrhagic malignant ascites, which were signs of advanced ovarian cancer. Similarly, bioluminescence signals were used to assess tumor progression. Treatment with LCMP@ALG efficiently inhibited the development of intra-abdominal tumors, with a more significant effect than that in LCPP@ALG group (Fig. 5B and C). Combination therapy of LCMP@ALG with anti-PD-1 exhibited slightly better tumor inhibition than LCMP@ALG administration alone. This result was further confirmed with tumor nodules in the abdominal cavity (Fig. 5D). As shown in Fig. 5E–G and Supporting information Fig. S18, LCMP@ALG and LCMP@ALG + anti-PD-1 inhibited the progression of hemorrhagic malignant ascites, exhibiting reduction in ascites volume and decrease in red blood cells in the ascites. Meanwhile, there were fewer metastatic tumors attached to the uterus, according to the images and weights of uterine appendages (Supporting information Fig. S19). Also, there were no significant changes in body weight, H&E staining of major organs and skin, and blood biochemistry parameters (Supporting information Figs. S20–S22), revealing that the vaccine LCMP@ALG had good biocompatibility. These results suggested that LCMP@ALG combined with anti-PD-1 therapy effectively induced a protective antitumor immune response.

**Figure 5:**
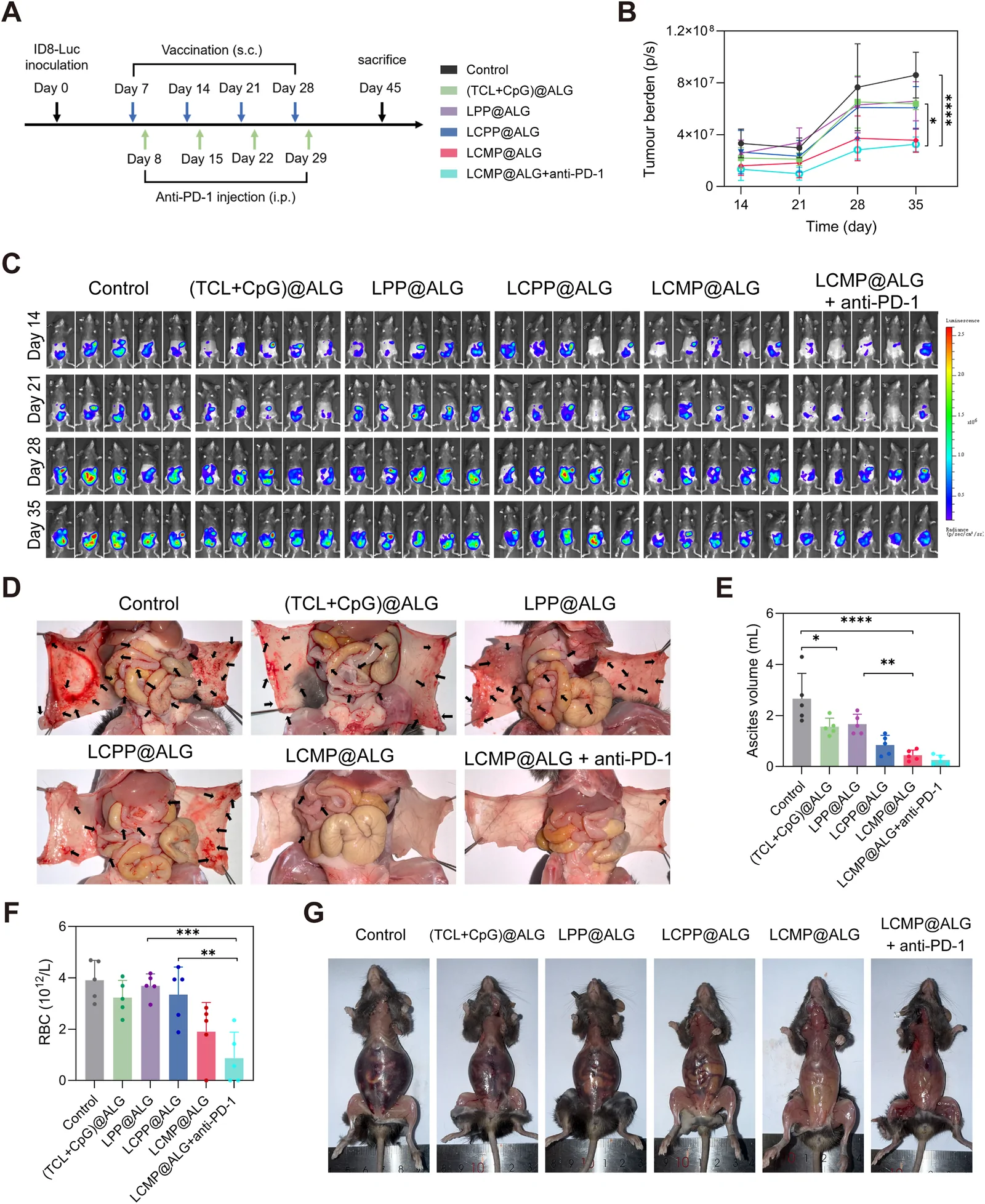
Inhibition of tumor progression by LCMP@ALG. (A) Treatment timeline for treating tumors. (B) Semi-quantification data and (C) *in vivo* bioluminescence imaging of ID8-Luc tumor-bearing mice (*n* = 5). (D) Representative images of tumor nodules after treatment with different formulations. (E) The volume of ascites after treatment with different formulations. (F) Red blood cells (RBC) count within ascites. (G) Representative images showing the development of peritoneal haemorrhagic ascites. Data are presented as mean ± SD (*n* = 5). ∗*P* < 0.05, ∗∗*P* < 0.01, ∗∗∗*P* < 0.001, ∗∗∗∗*P* < 0.0001, and “ns” stands for no significant difference.

### 3.5 Immune activation *in vivo*

To further understand the antitumor processes, the immune response in mice was examined. Firstly, the DCs maturation in LNs and spleens were determined. Compared with other groups, there were significantly upregulated expression of CD80, CD86 and MHC I in the LNs and spleens of LCMP@ALG and LCMP@ALG + anti-PD-1 groups, suggesting enhanced DCs maturation and antigen presenting (Fig. 6A–E, Supporting information Fig. S23). Meanwhile, activated B cells and plasma cells of the LCMP@ALG group were also promoted, revealing improved antigen presenting and antibody secreting abilities of B cells and their potential to activate antitumor humoral immunity (Fig. 6F and G).

**Figure 6:**
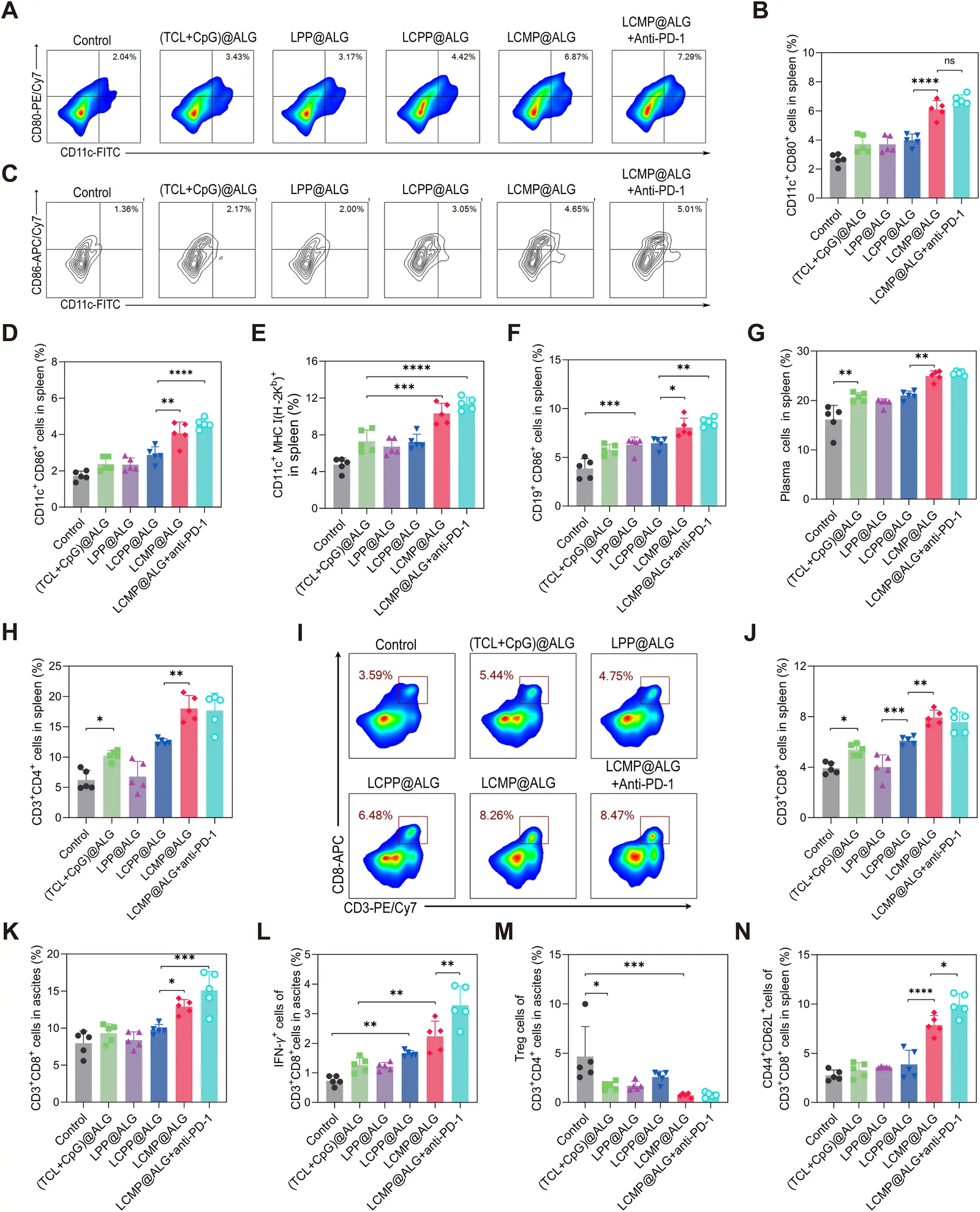
*In vivo* antitumor immune response. (A) Representative flow cytometry plots and (B) percentages of CD11c^+^CD80^+^ cells in the spleen of each group. (C) Representative flow cytometry plots and (D) percentages of CD11c^+^CD86^+^ cells in the spleen of each group. (E) Flow cytometry analysis of CD11c^+^ MHC Ⅰ^+^ cells in the spleen of each group. Flow cytometry analysis of (F) CD86^+^ B cells and (G) plasma cells of each group. (H) Flow cytometry analysis of CD4^+^ T cells of each group. (I) Representative flow cytometry plots and (J) percentages of CD8^+^ T cells in the spleen of each group. (K) percentages of CD8^+^ T cells in ascites of each group. (L) Flow cytometry analysis of IFN-*γ*-secreting CD8^+^ T cells of each group. (M) Flow cytometry analysis of Tregs of each group. (N) Flow cytometry analysis of CD8^+^ Tcm of each group. Data are presented as mean ± SD (*n* = 5). ∗*P* < 0.05, ∗∗*P* < 0.01, ∗∗∗*P* < 0.001, ∗∗∗∗*P* < 0.0001, and “ns” stands for no significant difference.

Maturation of DCs and activation of B cells would further contribute to the activation of T cells. Thus, the phenotype and abundance of T cells in LNs, spleens, and ascites were determined. LCMP@ALG increased the percentages of activated T cells (CD69^+^CD4^+^ T cells and CD69^+^CD8^+^ T cells) in the LNs (Supporting information Fig. S24). The CD8^+^ T cells and CD4^+^ T cells in the spleen were also upregulated by LCMP@ALG from 3.92% to 7.94% and 6.21% to 18.03%, respectively (Fig. 6H–J). And the percentages of CD8^+^CD25^+^ T cells were also significantly increased in the LCMP@ALG and LCMP@ALG + anti-PD-1 groups in the spleen (Supporting information Fig. S25). Meanwhile, in the ascites, LCMP@ALG and LCMP@ALG + anti-PD-1 treated mice presented higher abundance of CD8^+^ T cells and IFN-*γ*-secreting CD8^+^ T cells than mice in other groups (Fig. 6K and L). In addition, LCMP@ALG and LCMP@ALG + anti-PD-1 treated mice exhibited the lowest abundance of Tregs (Fig. 6M).

Whether LCMP@ALG could induce antitumor immune memory was further explored. The percentages of the Tcm (CD44^+^CD62L^+^CD4^+^ T cells and CD44^+^CD62L^+^CD8^+^ T cells) in the spleen of the LCMP@ALG group were 1.57- and 2.03-fold higher than those in LCPP@ALG group, respectively (Fig. 6N, Supporting information Fig. S26). Besides, the combination of LCMP@ALG and PD-1 antibody significantly increased Tcm levels.

Together, these results jointly verified that the vaccine with enhanced synergistic effects exhibited strong immunostimulatory ability, which induced DCs maturation, B cell differentiation, T cell activation, and antitumor immune memory to exert antitumor effects.

## 4 Conclusion

To address the constraint of antitumor immune response efficacy in the immunotherapy of ovarian cancer, we exploited the whole-cell tumor antigen and nanotechnology to generate a versatile mannosylated nanovaccine LCMP@ALG. When compared to free TCL mixed with adjuvants and conventional PEG modification nanovaccines, mannosylated nanovaccines induced stronger antitumor immunity and demonstrated improved antitumor effect and survival rates.

LCMP@ALG was an *in situ* hydrogel cancer vaccine that integrated multiple rational design strategies to coordinate the antitumor immune cascade. LCMP@ALG provided many advantages over previously reported approaches, including broad-spectrum tumor antigens, efficient uptake of APCs, multivalent presentation of TLRs, activation of both cellular and humoral immunity, strong antitumor effectiveness without evidence of toxicity, and simplified manufacture for wider availability. The ALG hydrogel ensured specific and sustained antitumor immune response through continuous release of nanovaccine LCMP. LCMP was effectively captured by APCs and displayed superior LNs accumulation through ligand-mediated active targeting capacity. By co-delivery of TCL and adjuvants, LCMP significantly enhanced DCs maturation and B cell differentiation, which further ignited robust B cells and T cells immune response. In particular, LCMP@ALG offered the multitargeting TLR co-stimulatory effect (CpG-induced TLR9 and MAN-induced TLR4), which potentiated the efficient maturation towards Th1-polarized immune responses. While TLR agonists were widely used as adjuvants, the combination of multiple TLR pathways served to broaden the immune response profile^57^^,^^58^. Dual TLR engagement not only enhances DCs function but also directly stimulates B cells and amplifies antibody production, which were essential for maintaining tumor site homeostasis and subsequently triggering adaptive immunity^47^^,^^59^.

Clinical trials combining antitumor vaccines with immune checkpoint treatment had shown encouraging results in several cancers^60^, ^61^, ^62^. Our results demonstrated the synergy of antitumor nanovaccines with PD-1 antibody therapy, which presented substantial antitumor effects, including considerable tumor growth suppression and significant reduction of malignant hemorrhagic ascites. The tumor immunosuppressive microenvironment was remodeled into an immune activation state with an increase in the proportion of mature DCs, significant activation of B cells, enhanced T cell infiltration, decreased Tregs, and development of long-term immunological memory. Among them, several immune markers in LCMP@ALG + anti-PD-1 group did not show statistically significant improvements compared to LCMP@ALG alone. This may be attributed to the relatively small sample size, as well as the effect of PD-1 blockade primarily exerted within the tumor microenvironment. Nevertheless, the combination therapy consistently enhanced overall antitumor efficacy, suggesting a trend toward synergistic benefit.

Our vaccine platform might provide a clinical alternative to treatments involving autologous DCs, which are linked with complex, time-consuming, and costly procedures. By using TCL as the antigen source, LCMP@ALG not only bypassed the current challenges in identifying neoantigens but also tackled tumor heterogeneity by presenting both tumor-associated antigens and potential neoantigens. This approach decreased the risk of tumor immune escape triggered by defined antigenic peptides. TCL can be rapidly prepared using either surgically resected tumor tissue or ascites fluid, both of which are readily accessible in clinical practice^28^. In addition, vaccines based on TCL may have great potential to provide a modality to generate universal antitumor protection^63^. While the present platform's antitumor efficiency was encouraging, the findings also highlighted the need for more research to optimize the immunogenicity of TCL. It has been reported that methods such as heat shock, oxidative modification, and extraction of water-insoluble antigens can improve the immunogenicity of TCL^64^, ^65^, ^66^.

In summary, our study describes the design of a function-oriented nanovaccine approach LCMP@ALG, which may be applicable to the efficient co-delivery of antigens and adjuvants, demonstrating a multi-pronged design strategy for immunotherapy of ovarian cancer.

## Author contributions

Jia Yan: Conceptualization, Investigation, Methodology, Writing – original draft. Ruyi Lin: Conceptualization, Investigation, Methodology, Writing – review & editing. Qiyu Liu: Investigation, Methodology, Writing – review & editing. Yatong Zhang: Software, Methodology. Hao Chen: Investigation and Methodology. Yujun Song: Methodology and Software. Yulong Shi: Methodology and Software. Fan Tong: Methodology, Writing – review & editing. Yuan Li: Conceptualization, Supervision, Funding acquisition.

## Conflicts of interest

There are no conflicts to declare.

## Appendix A Supporting information

The following is the Supporting information to this article.
