# Supplementary material for: In situ hydrogel nanovaccine based on whole-cell tumor antigen efficiently activates adaptive immunity for the prevention and treatment of ovarian cancer
Source: Acta Pharm Sin B. 2026 Jul 24;16(11):7573–88. doi: 10.1016/j.apsb.2026.07.030 (PMC13730230; doi:10.1016/j.apsb.2026.07.030)
Supplement: Supplementary file 1 [file mmc1.pdf]

Supporting Information for

Original article

In situ hydrogel nanovaccine based on whole-cell tumor antigen efficiently activates adaptive immunity for the prevention and treatment of ovarian cancer

Jia Yan[a,b,†], Ruyi Lin[b,†], Qiyu Liu[a,†], Yatong Zhang[b], Hao Chen[b], Fan Tong[b], Yujun Song[b], Yulong Shi[b], Yuan Li[a,*]

[a]*Department of Obstetrics and Gynecology, Third Hospital of Peking University, Beijing 100191, China*

[b]*Key Laboratory of Drug Targeting and Drug Delivery Systems, West China School of Pharmacy, Sichuan University, Chengdu 610041, China*

Received 7 March 2026; received in revised form 22 April 2026; accepted 10 June 2026

[*]Corresponding author.

E-mail address: yuanli@bjmu.edu.cn (Yuan Li).

[†]These authors made equal contributions to this work.

Supporting figures

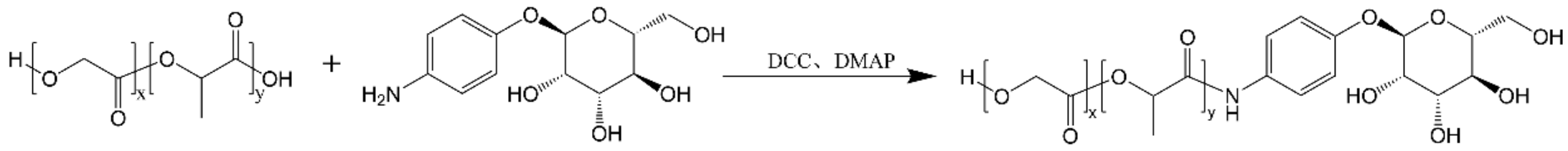

Scheme S1 Synthetic route of MAN-PLGA.

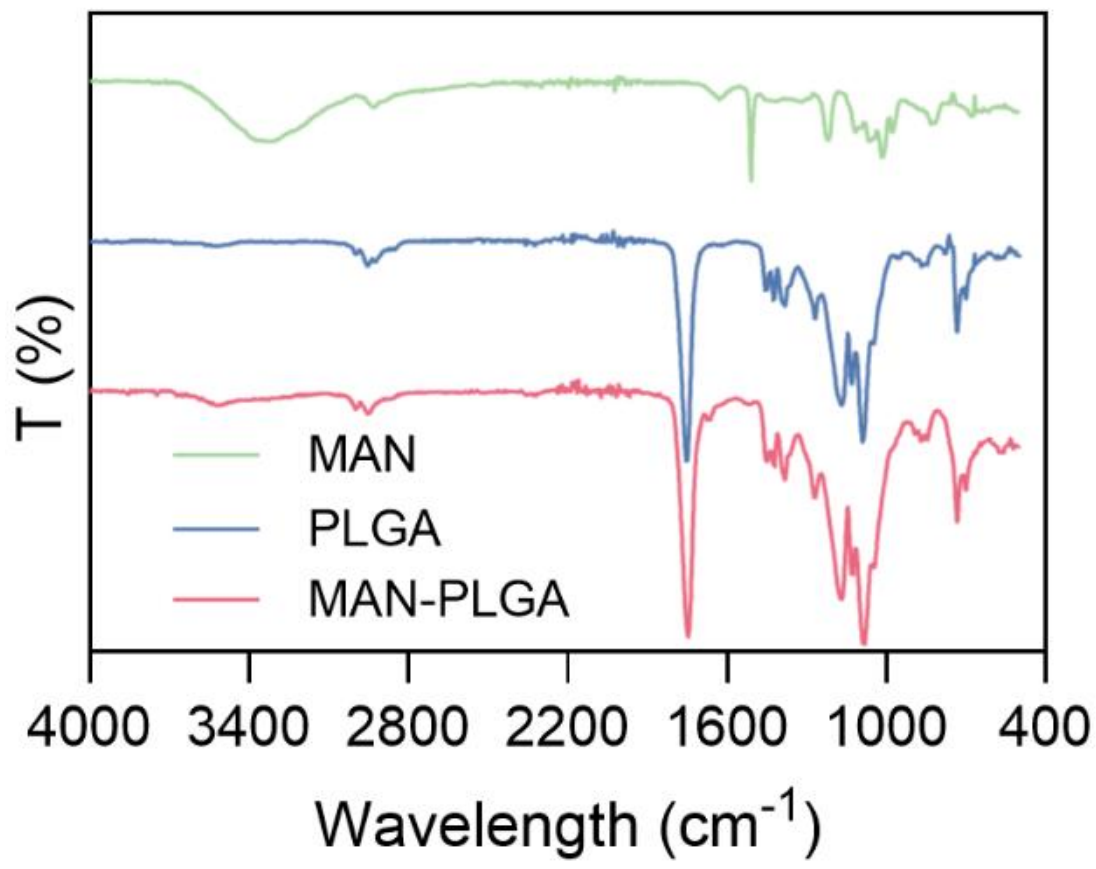

Figure S1 FTIR absorption spectra of MAN, PLGA and MAN-PLGA groups.

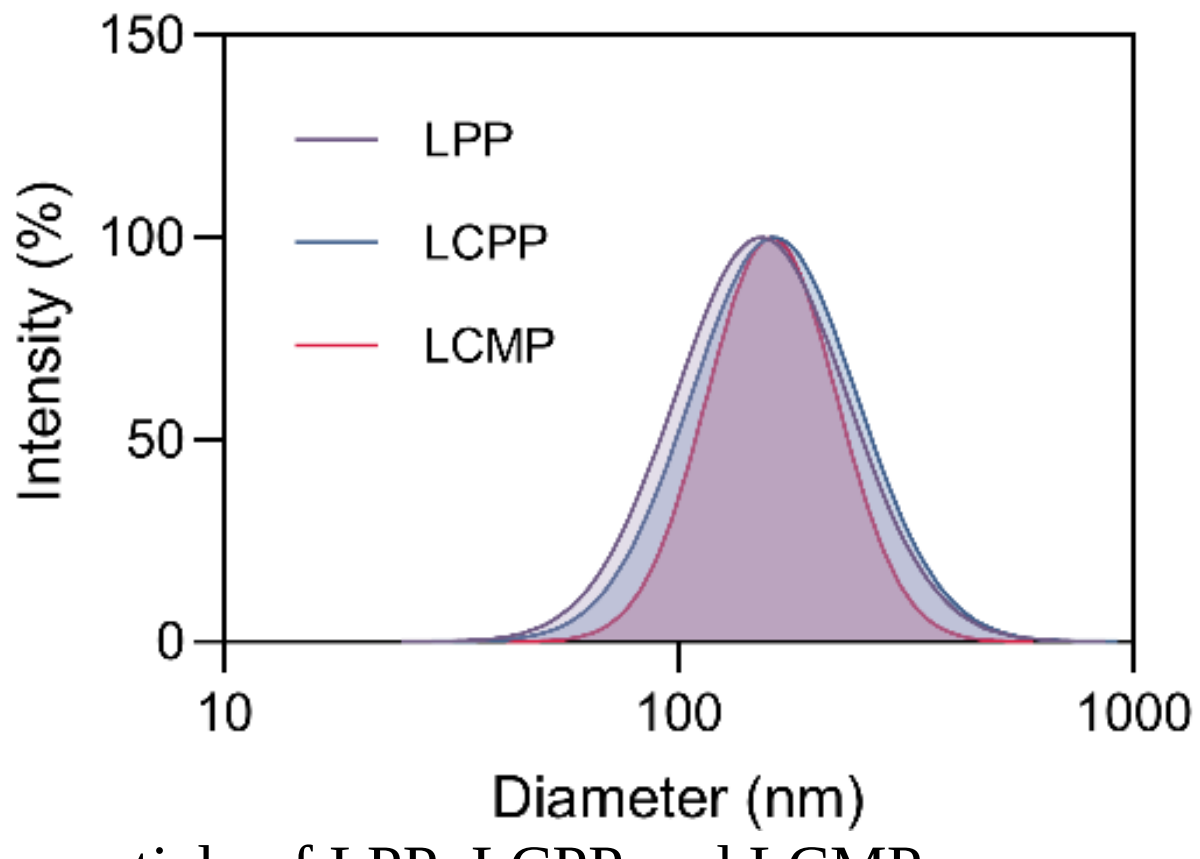

Figure S2 Hydration particle of LPP, LCPP and LCMP.

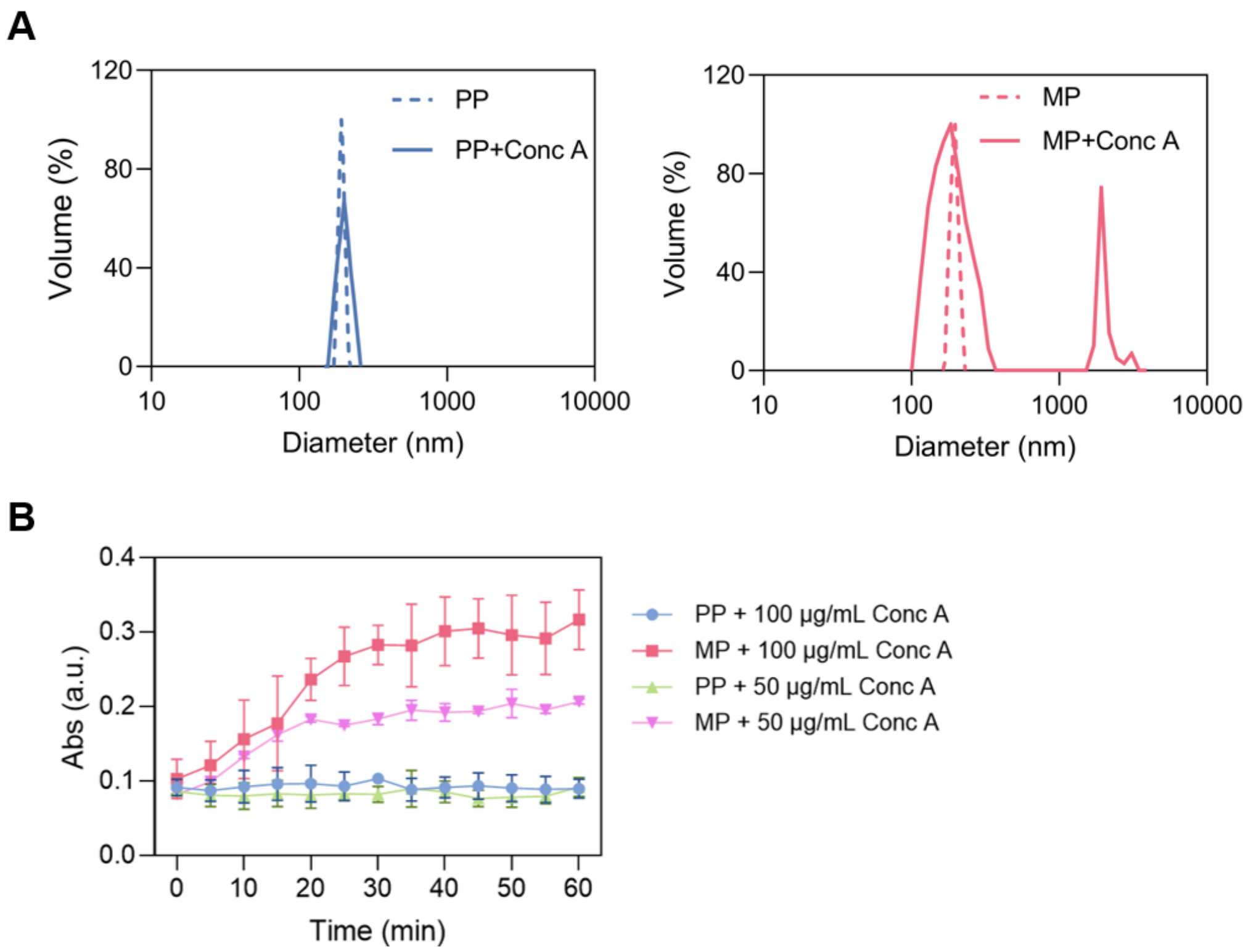

Figure S3 (A) Particle aggregates detected by DLS after incubation of Conc A with PP (left) and MP (right). (B) The turbidity of PP and MP after incubation of Conc A at different concentrations.

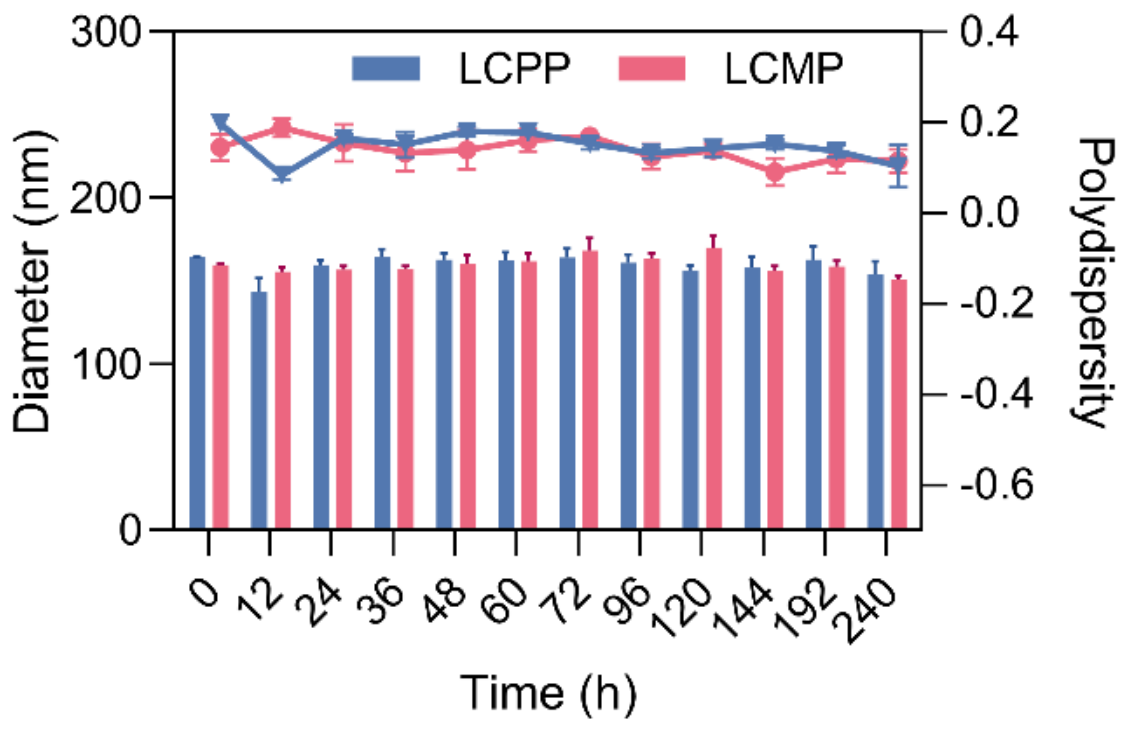

Figure S4 The stability of LCPP and LCMP at 4 °C (n = 3).

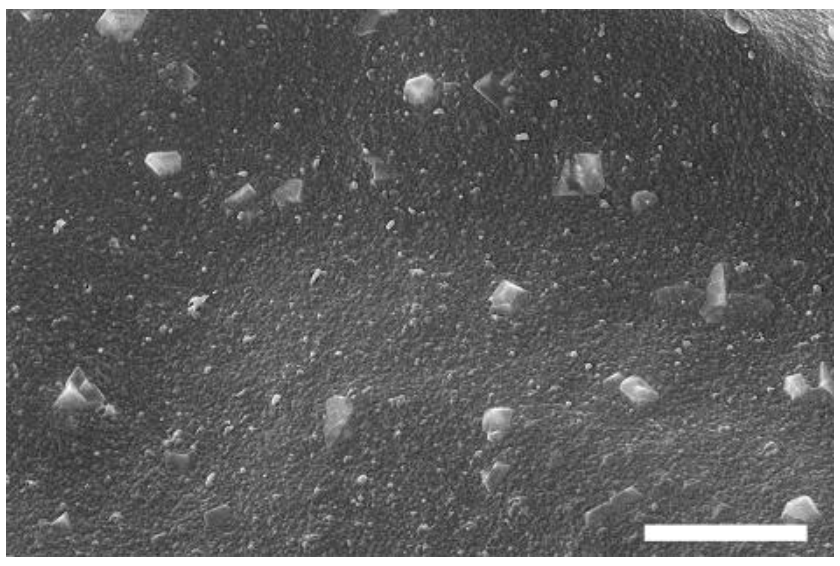

Figure S5 SEM image of LCMP@ALG. Scale bar = 5 μm.

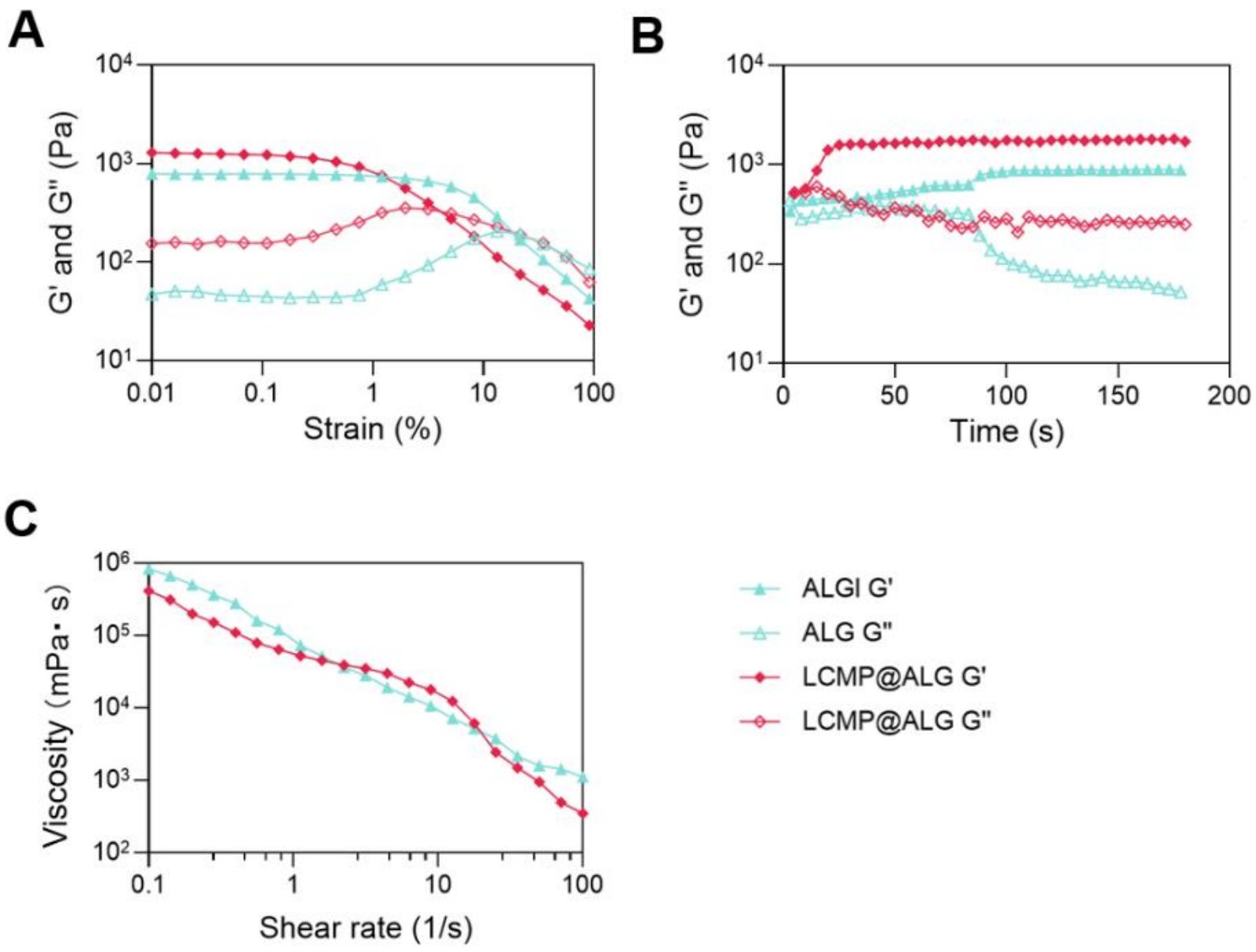

Figure S6 The variation of hydrogel modulus with (A) pressure and (B) time. (C) Hydrogel viscosity *versus* shear rate.

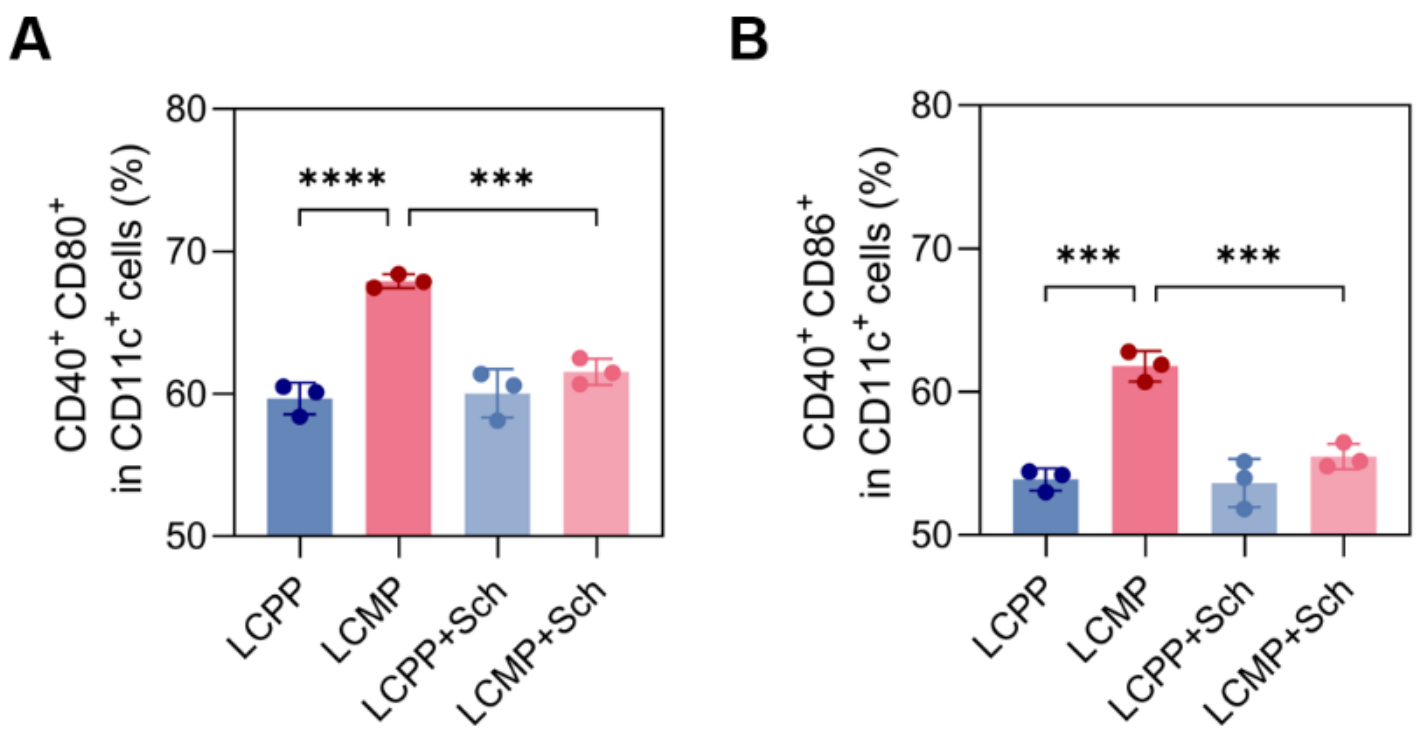

Figure S7 Flow cytometry analysis of (A) $CD40^{+}CD80^{+}$ cells and (B) $CD40^{+}CD86^{+}$ cells in BMDCs treated with different formulations for 24 h. ****P* < 0.001, *****P* < 0.0001.

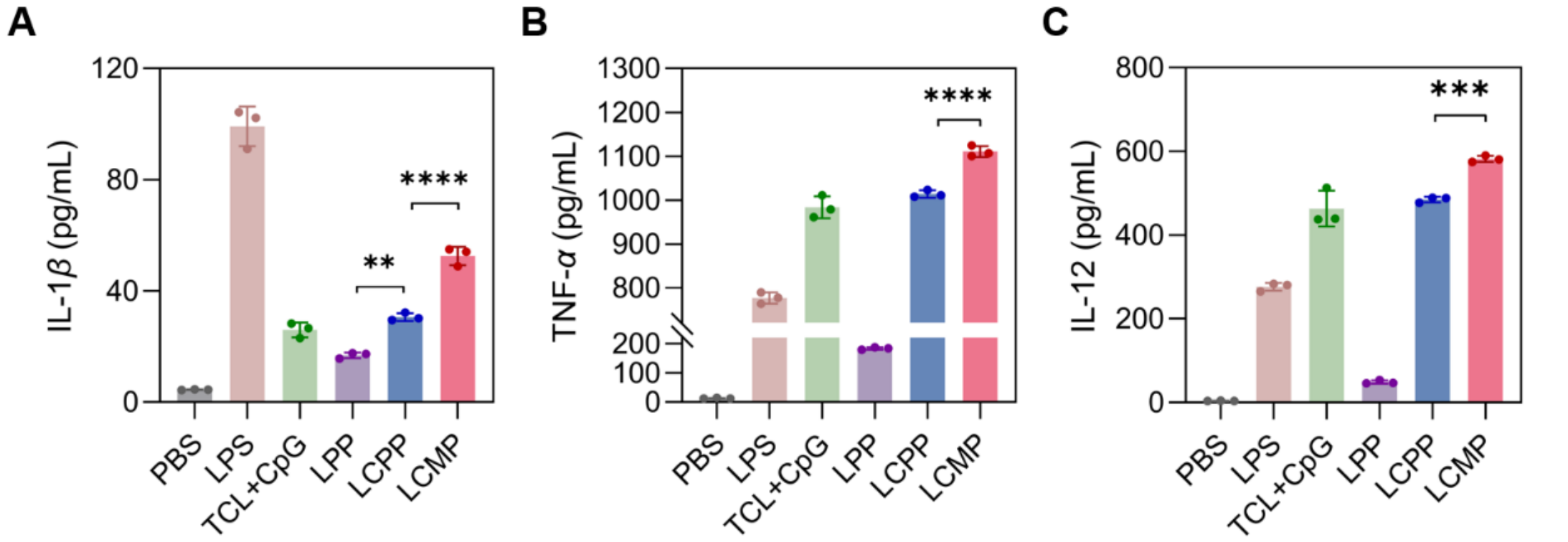

Figure S8 Expression of (A) IL-1β, (B) TNF-α, and (C) IL-12 by BMDCs. **$P < 0.01$, ***$P < 0.001$, ****$P < 0.0001$.

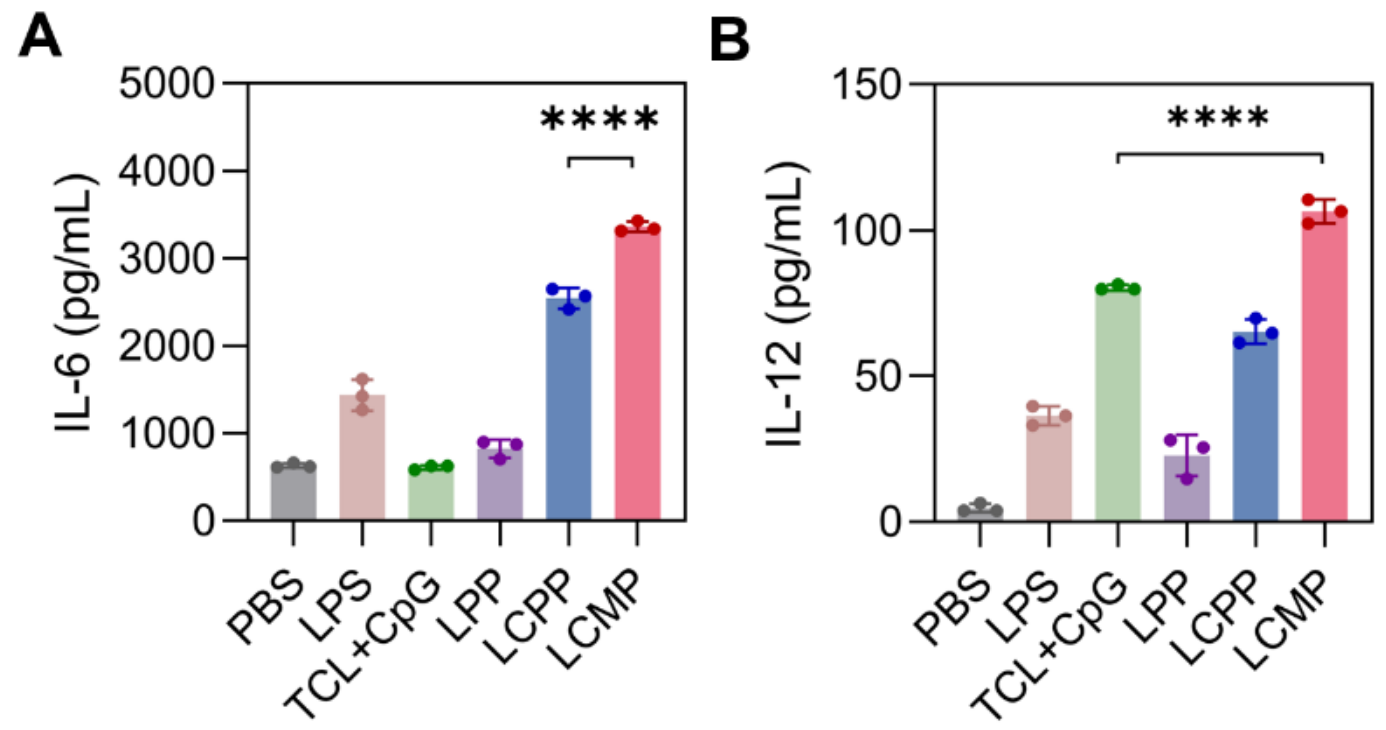

Figure S9 (A) IL-6 and (B) IL-12 release from lymphocytes after co-culture with BMDCs for 72 h. ****$P < 0.0001$.

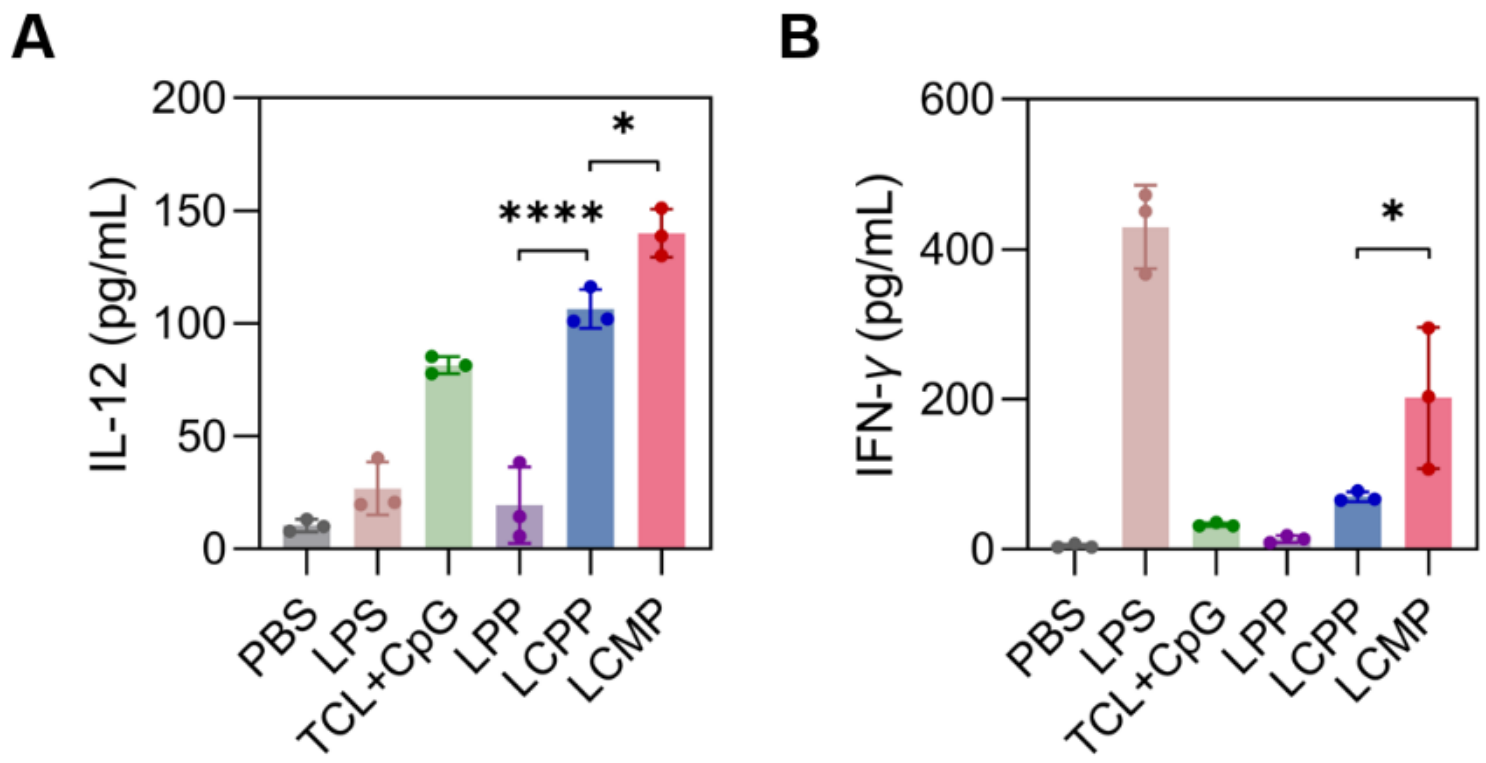

Figure S10 (A) IL-12 and (B) IFN-γ release from lymphocytes treated with different formulations for 48 h. *$P < 0.05$, ****$P < 0.0001$.

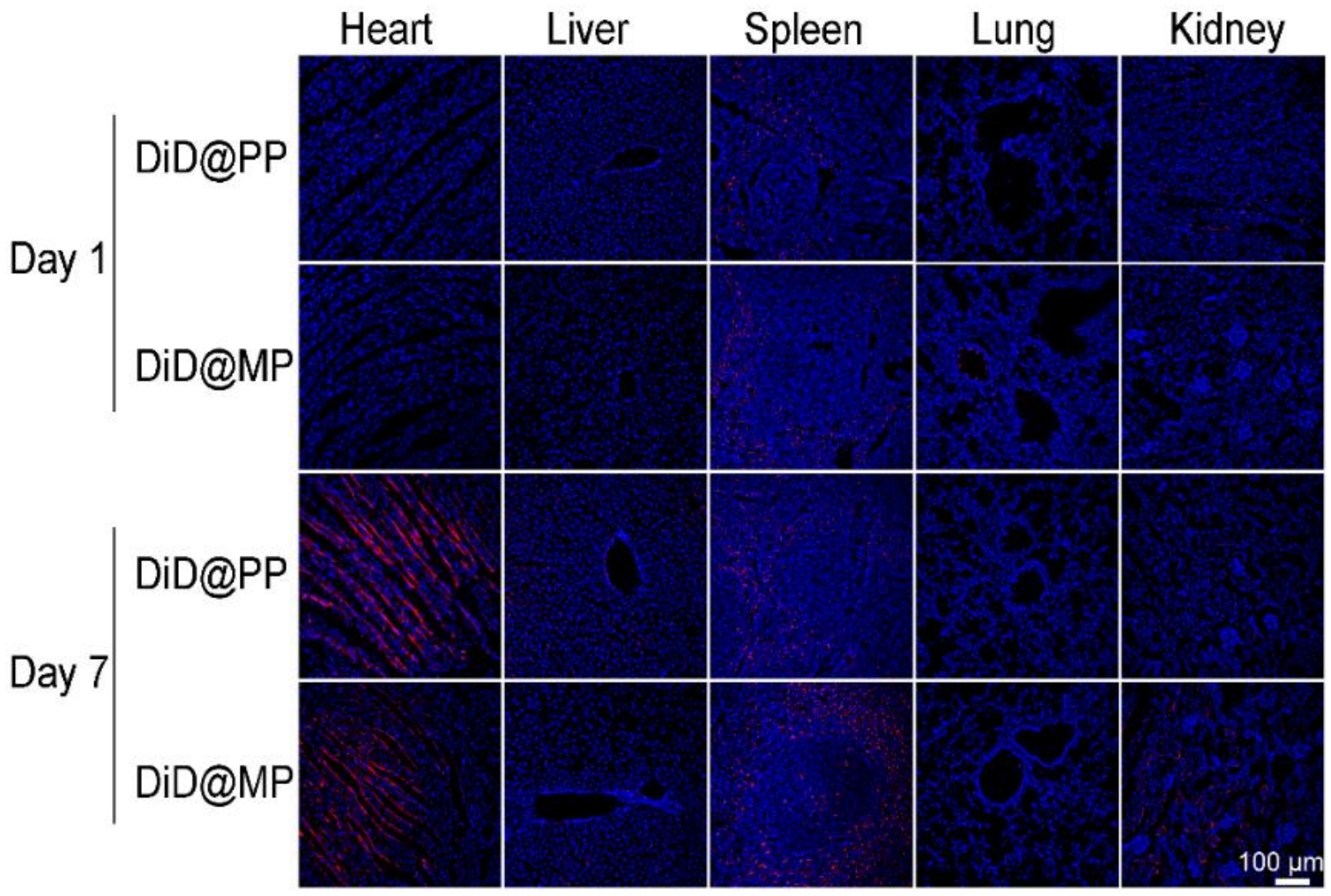

Figure S11 Fluorescent images of frozen sections of main organs. Scale bar = 100 μm.

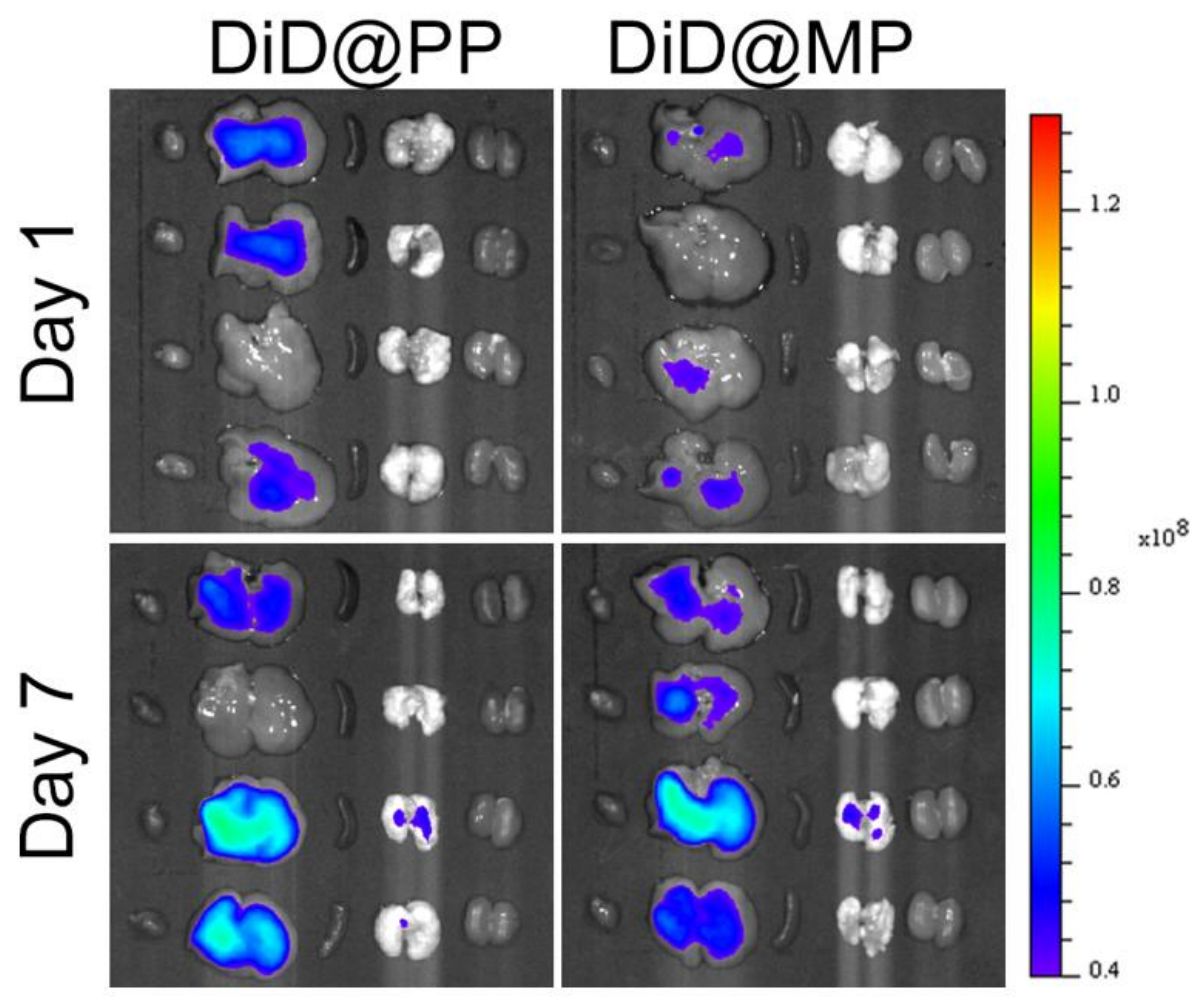

Figure S12 IVIS images of main organs.

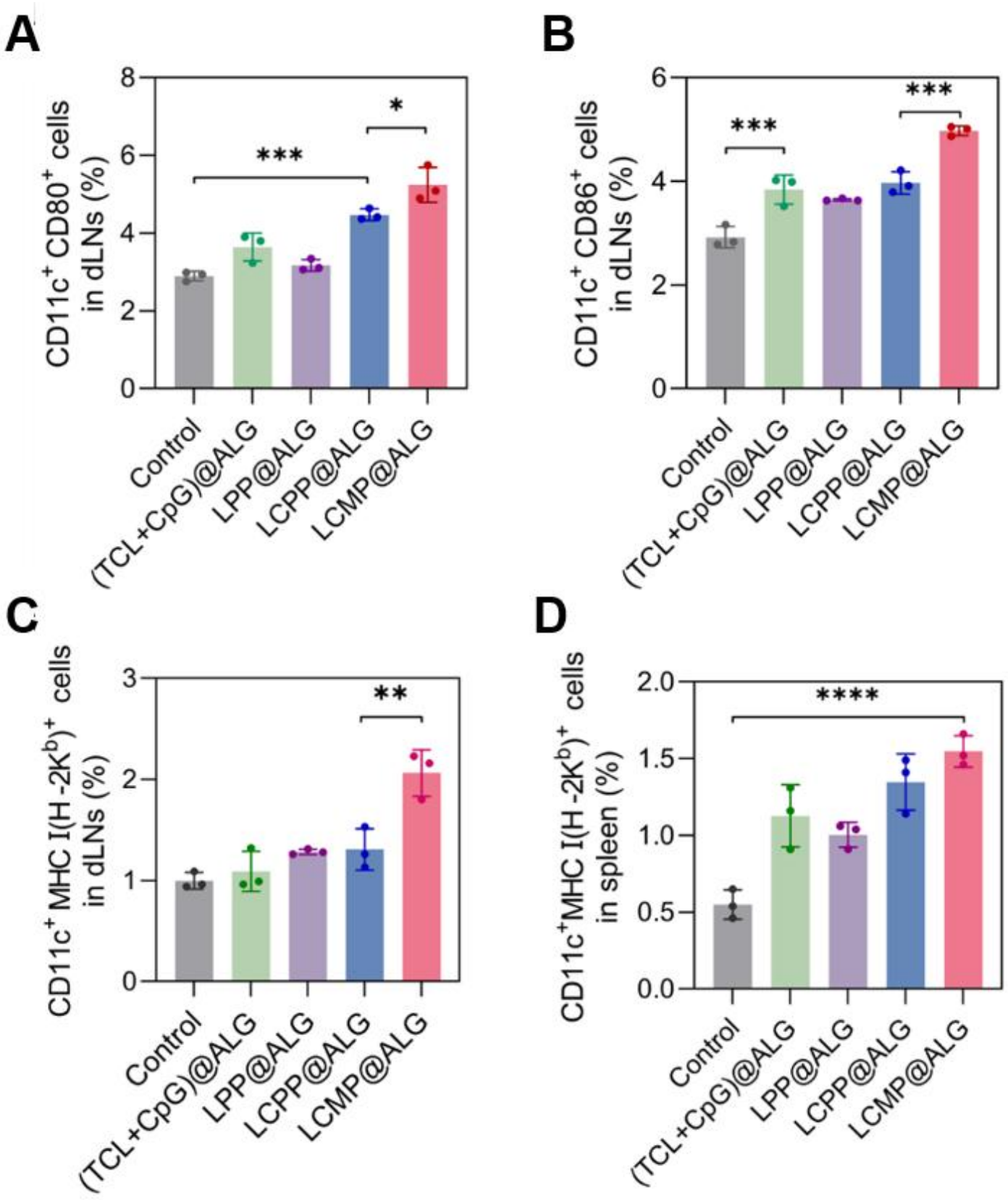

Figure S13 Percentages of (A) $CD11c^{+}CD80^{+}$, (B) $CD11c^{+}CD86^{+}$, and (C) $CD11c^{+}$MHC I^{+} cells in dLNs 2 days after vaccination. Percentages of (D) $CD11c^{+}$MHC I^{+} cells in the spleen 2 days after vaccination. $^{**}P < 0.01$, $^{****}P < 0.0001$.

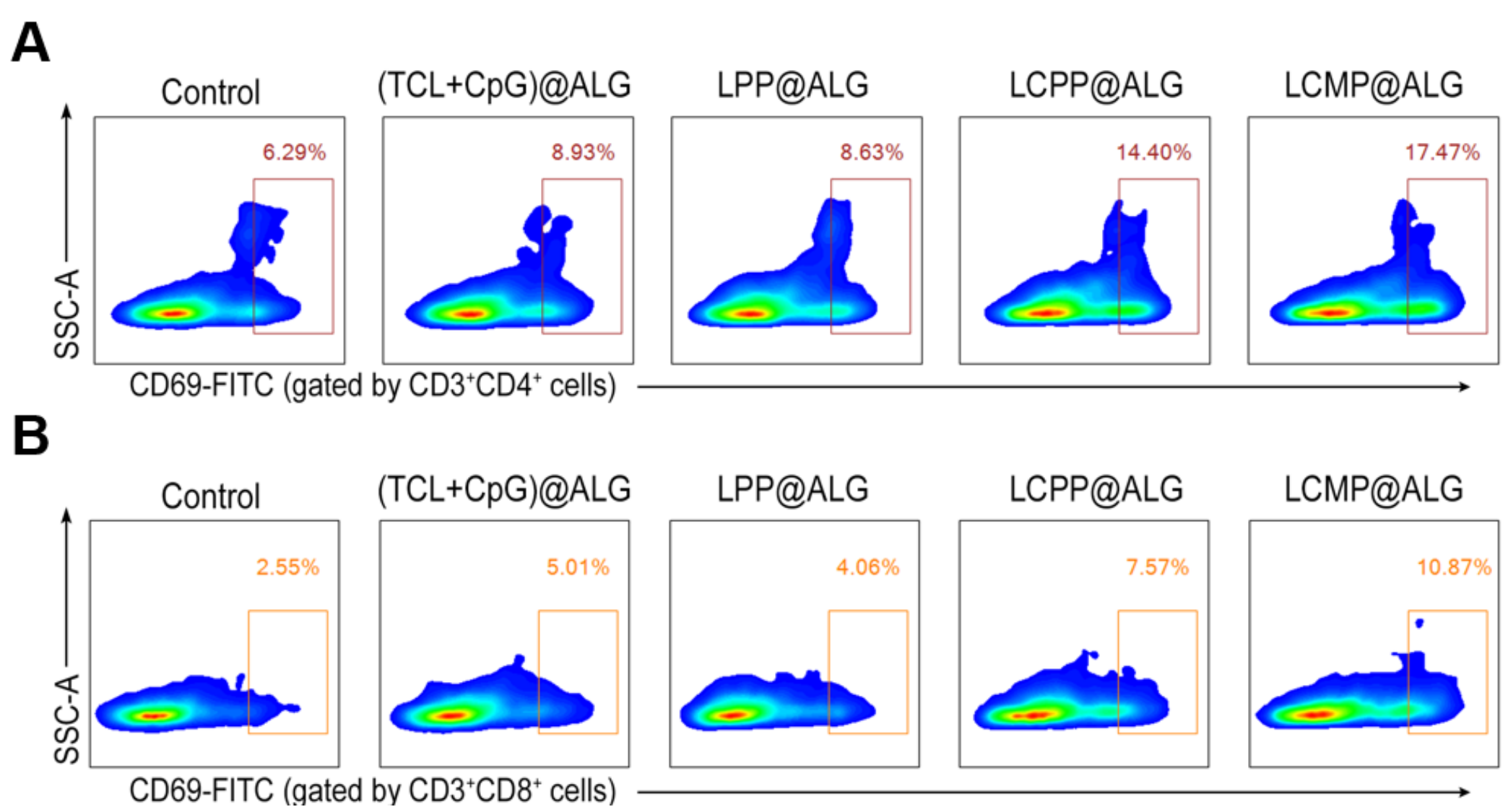

Figure S14 Representative images of (A) $CD69^{+}CD4^{+}$ T cells and (B) $CD69^{+}CD8^{+}$ T cells in the spleen 2 days after vaccination.

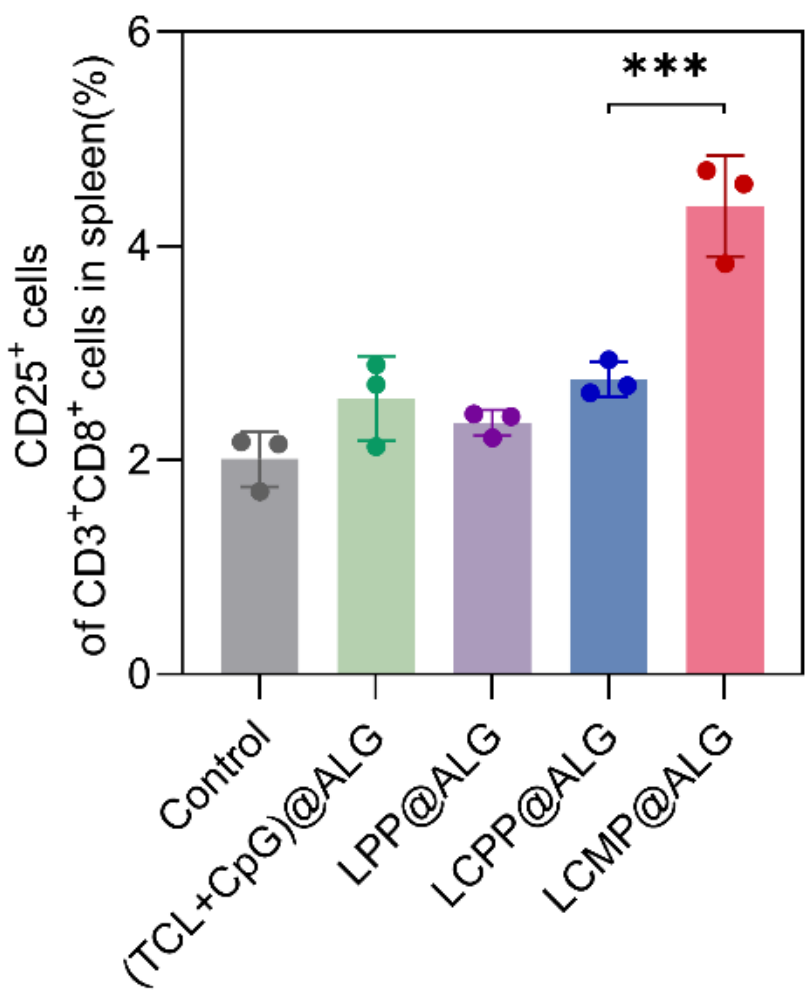

Figure S15 Flow cytometry analysis of CD25^{+}CD8^{+} cells in spleens *in vivo* 2 days after treatment (n = 3). ***P < 0.001.

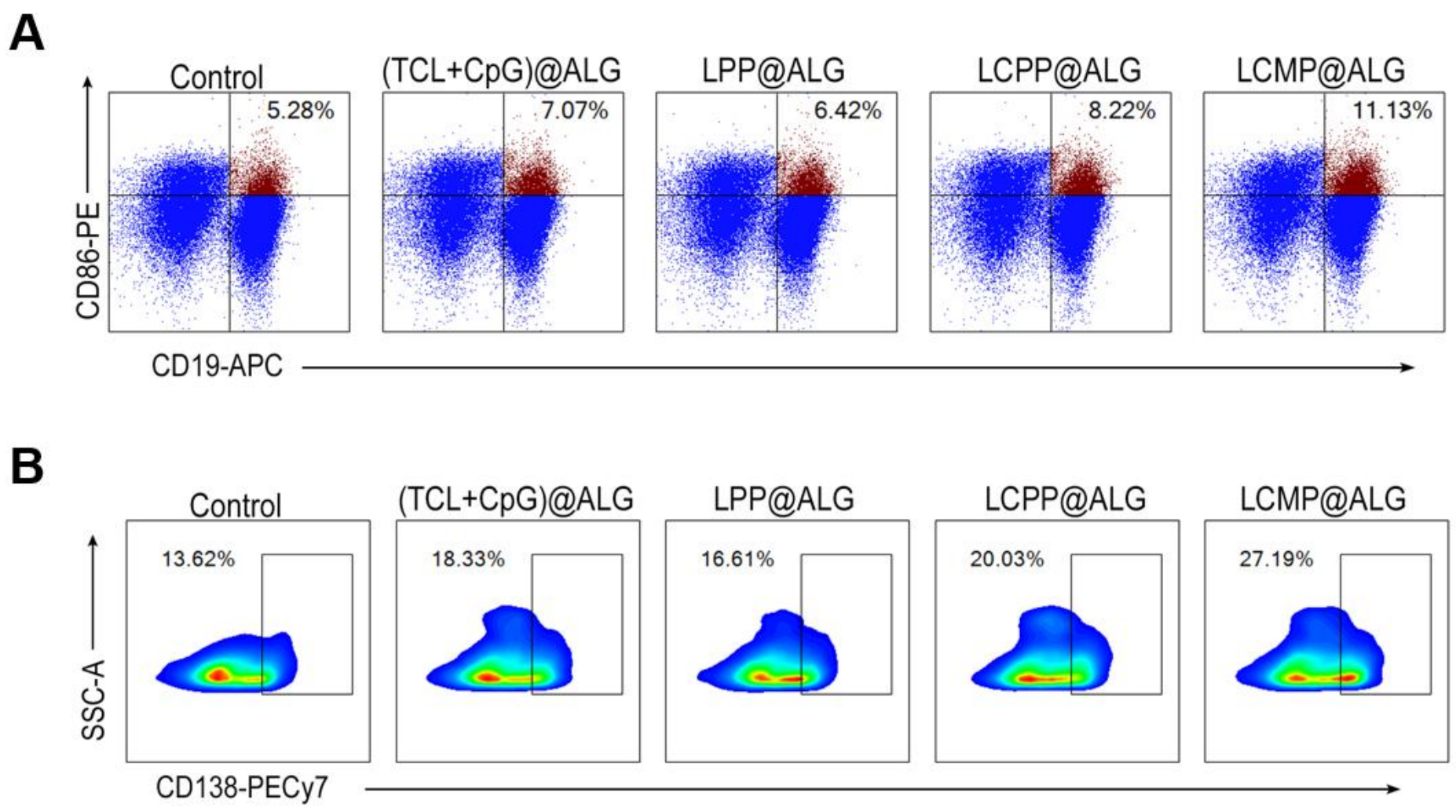

Figure S16 Representative images of (A) CD86^{+} B cells and (B) plasma cells in the spleen 2 days after vaccination.

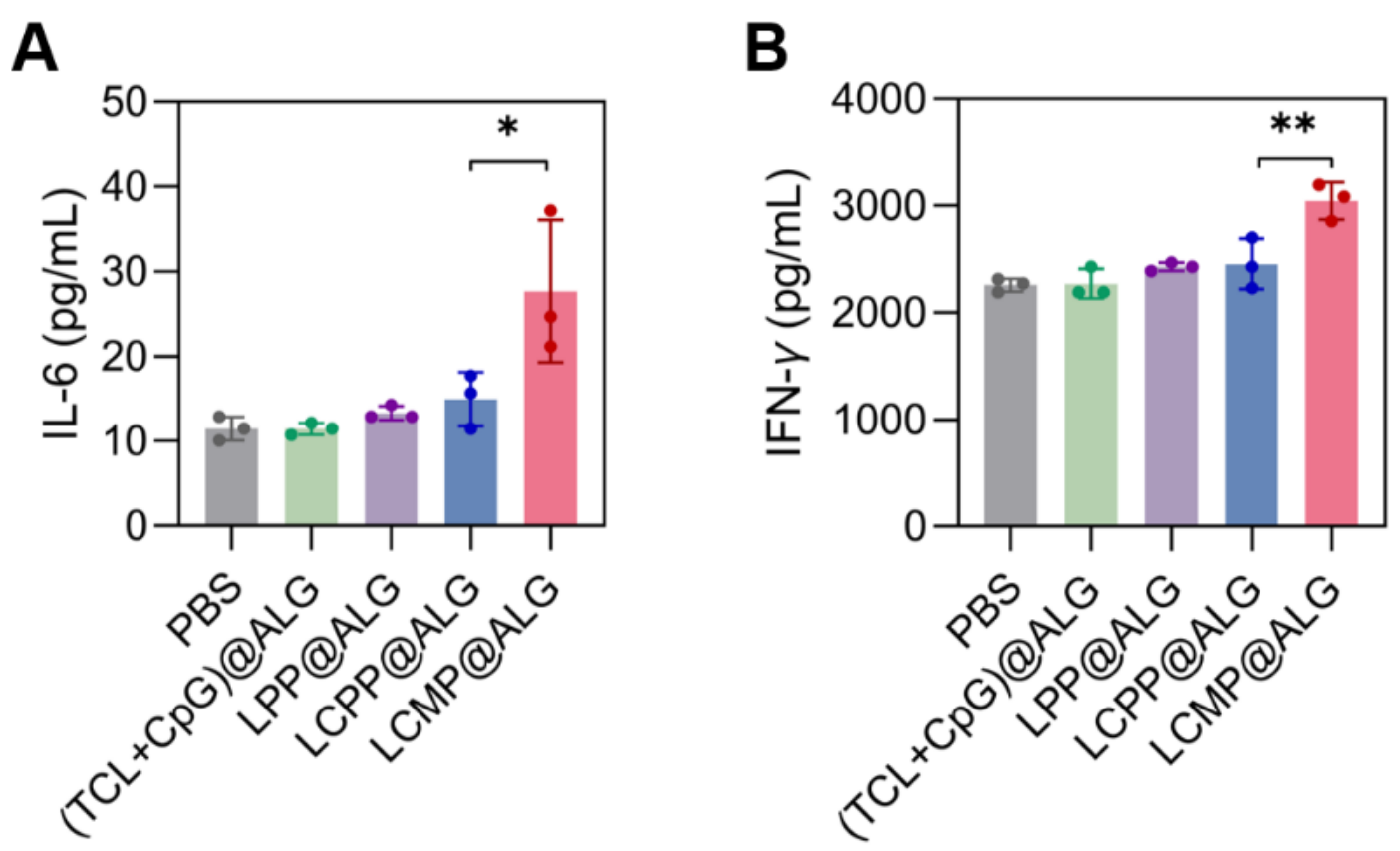

Figure S17 (A) IL-6 and (B) IFN-γ levels in the spleen, as detected by ELISA. **P* < 0.05, ***P* < 0.01.

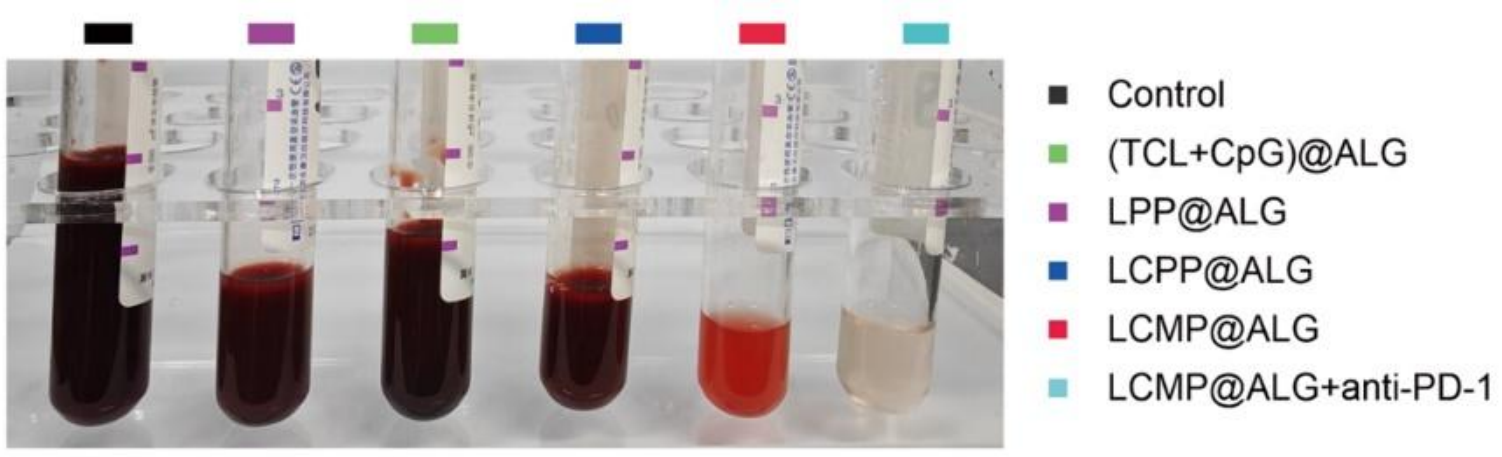

Figure S18 Representative images of ascites after treatment with different formulations.

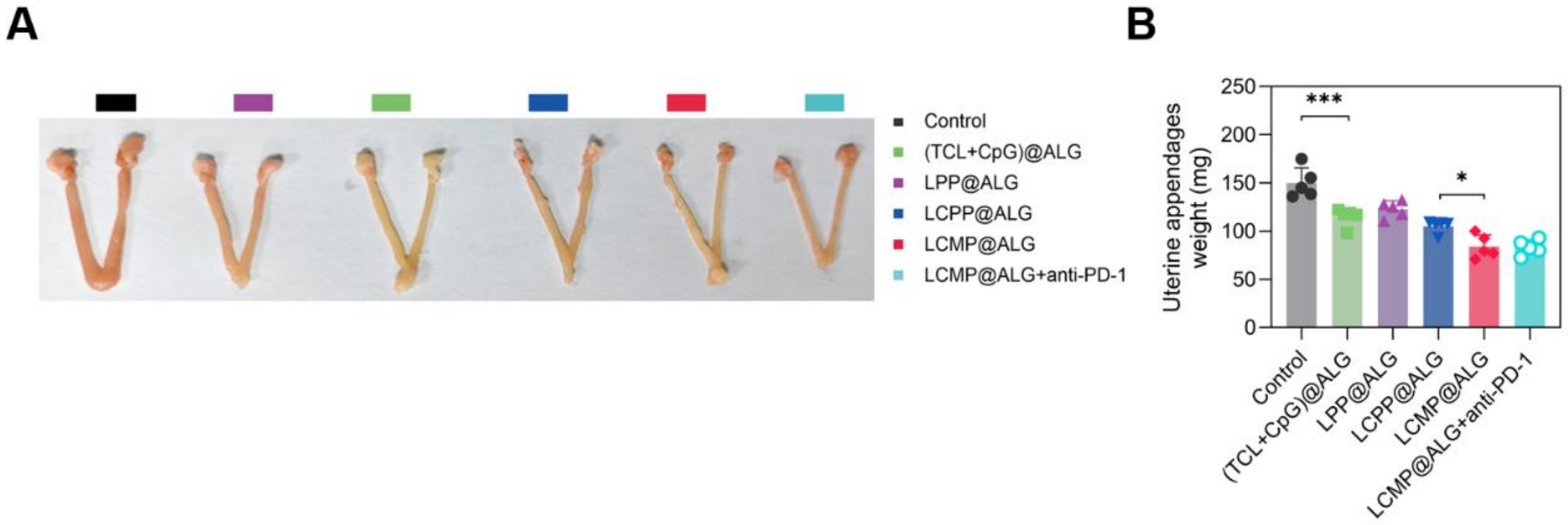

Figure S19 (A) Representative images of uterine appendages. (B) Uterine appendage weights after treatment with different formulations. *P* < 0.05, ***P* < 0.001.

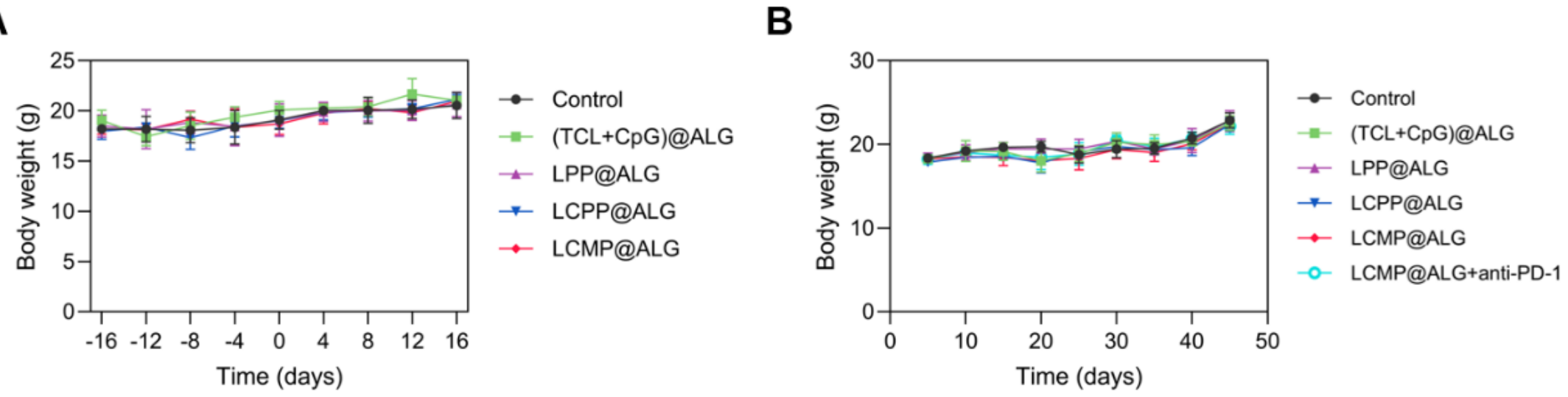

Figure S20 The body weight of mice in (A) prophylactic and (B) therapeutic experiments.

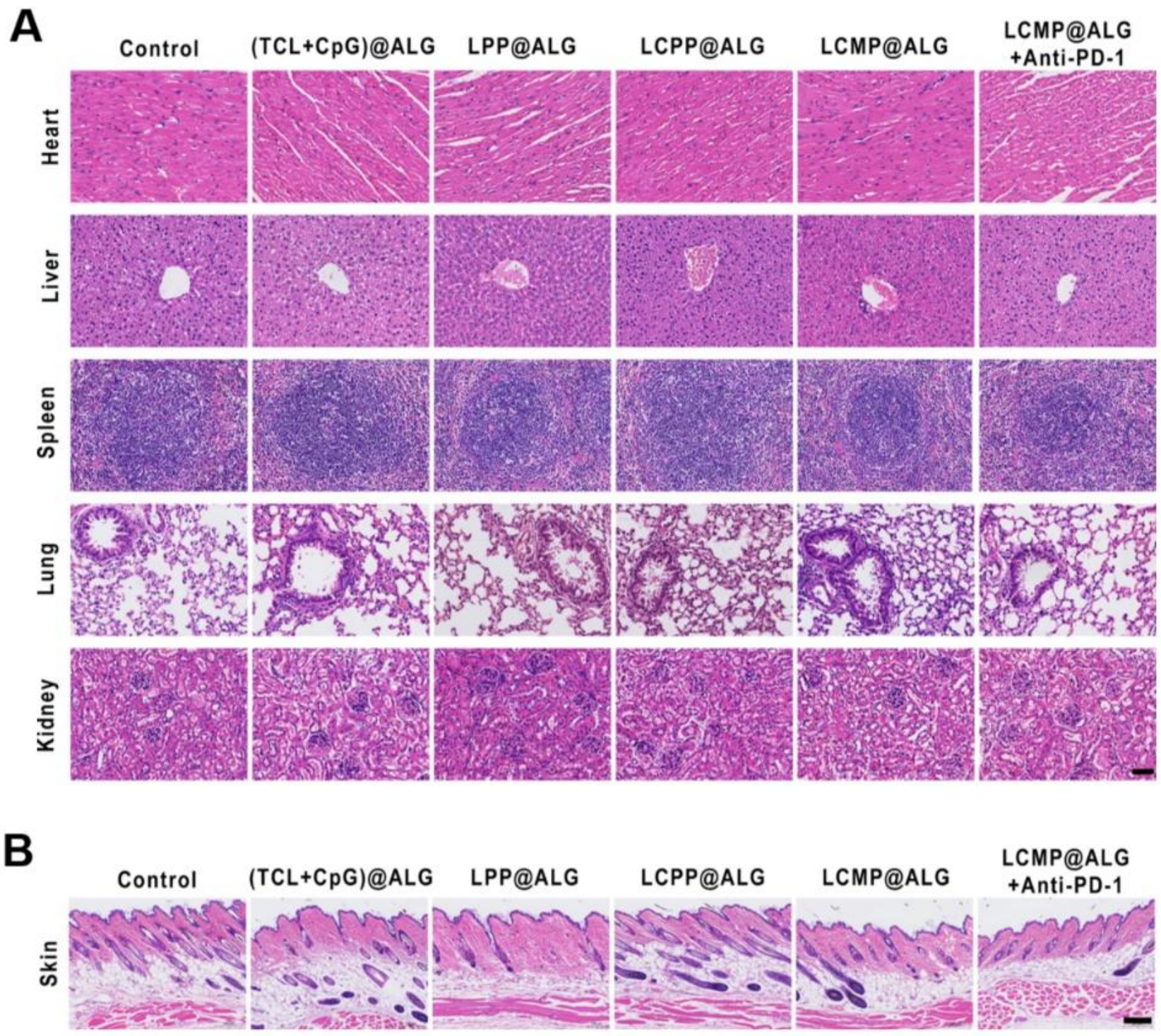

Figure S21 (A) Representative H&E-stained images of major organs post-treatment. Scale bar = 50 μm. (B) Representative H&E-stained images of skin at the administration site post-treatment. Scale bar = 200 μm.

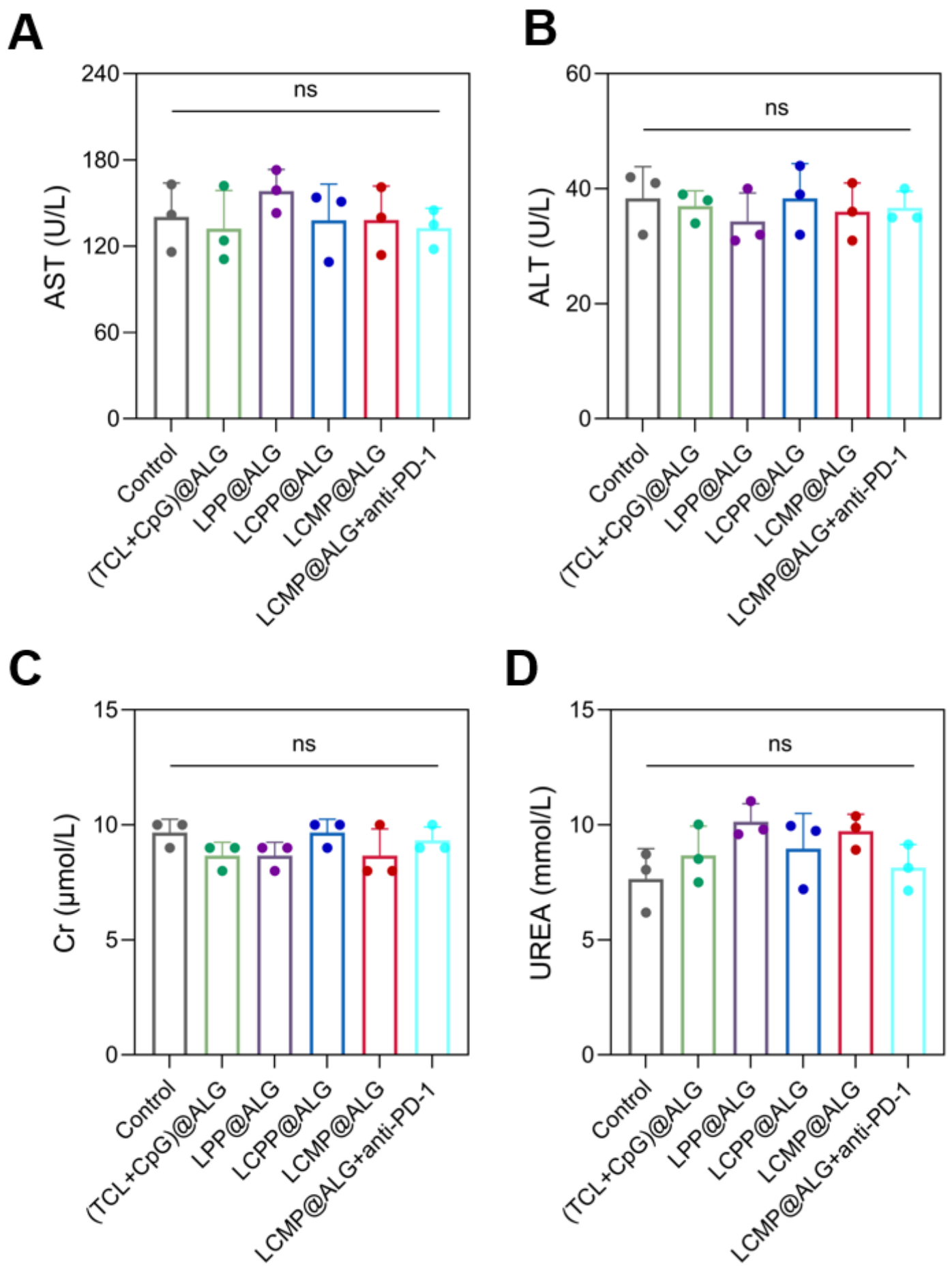

Figure S22 Serum biochemistry parameter analysis including (A) AST, (B) ALT, (C) Cr and (D) UREA. "ns" stands for no significant difference.

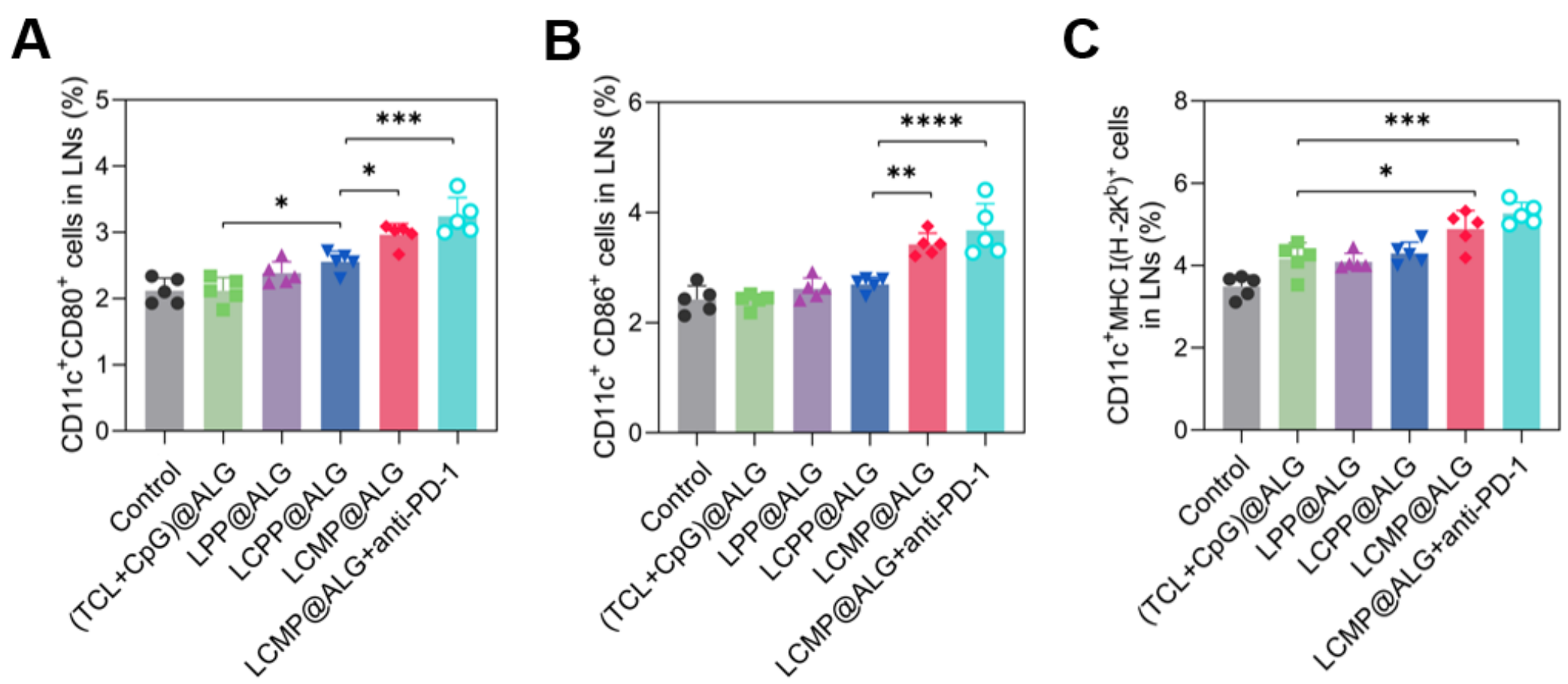

Figure S23 Flow cytometry analysis of (A) $CD11c^{+}CD80^{+}$ cells, (B) $CD11c^{+}CD86^{+}$ cells, and (C) $CD11c^{+}MHC\ I^{+}$ cells in LNs of each group. **P* < 0.05, ***P* < 0.01, ****P* < 0.001, *****P* < 0.0001.

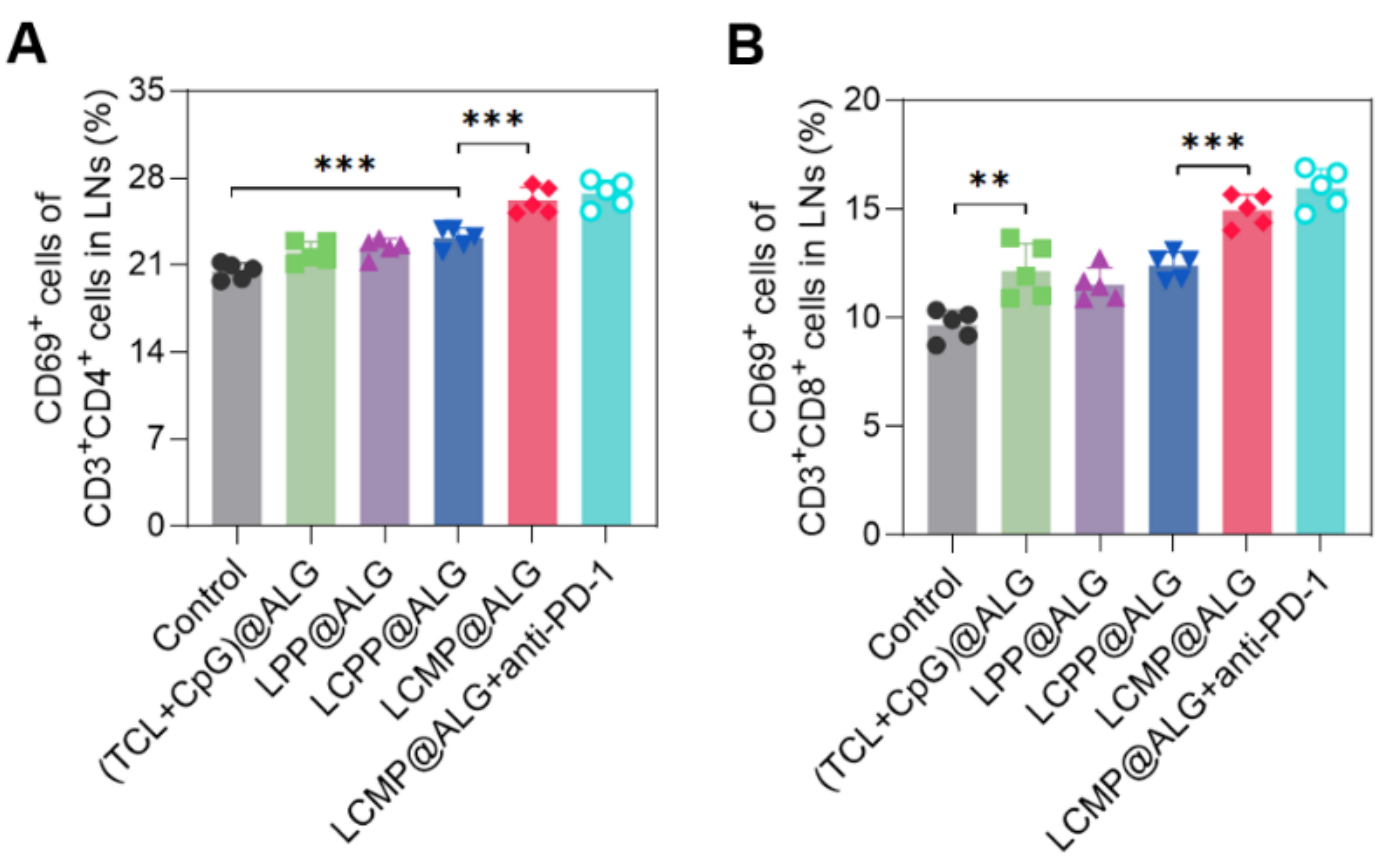

Figure S24 Flow cytometry analysis of (A) $CD69^{+}CD4^{+}$ T cells and (B) $CD69^{+}CD8^{+}$ T cells in LNs. $^{**}P < 0.01$, $^{***}P < 0.001$.

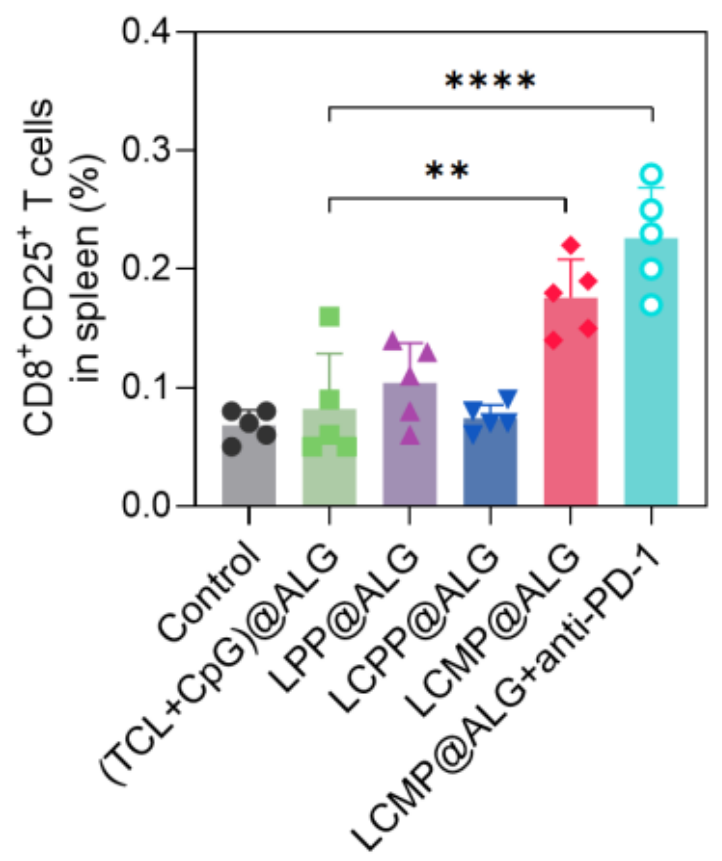

Figure S25 Flow cytometry analysis of $CD25^{+}CD8^{+}$ cells in spleen. $^{**}P < 0.01$, $^{****}P < 0.0001$.

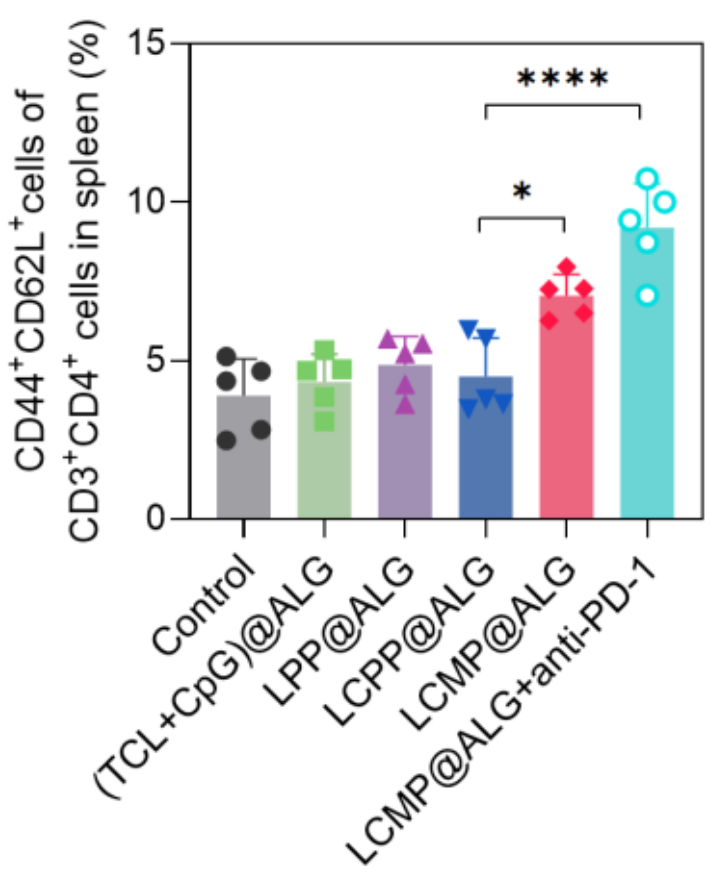

Figure S26 Flow cytometry analysis of $CD3^{+}CD4^{+}CD44^{+}CD62L^{+}$ cells in the spleen. $^{*}P < 0.05$, $^{****}P < 0.0001$.

Supporting tables

Table S1 Screening the dosage of TCL.

Dosage of TCL (mg)	Diameter (nm)	PDI	Loading capacity (%)	Encapsulation efficiency (%)
0.25	159.8 ± 1.8	0.195 ± 0.014	2.77 ± 0.26	44.25 ± 4.21
0.50	158 ± 3.2	0.132 ± 0.023	4.21 ± 0.27	36.19 ± 2.35
0.75	158.7 ± 3.1	0.159 ± 0.006	5.84 ± 0.55	25.17 ± 2.36
1.00	162.4 ± 4.5	0.123 ± 0.048	5.98 ± 0.45	17.14 ± 1.28

Data are presented as mean ± SD, *n* = 3.

Table S2 Screening the dosage of CpG.

Dosage of CpG (μg)	Diameter (nm)	PDI	CpG		TCL	
			Loading capacity (%)	Encapsulation efficiency (%)	Loading capacity (%)	Encapsulation efficiency (%)
12.5	182.6 ± 8.5	0.215 ± 0.013	0.12 ± 0.00	20.28 ± 0.28	3.75 ± 0.43	15.75 ± 1.80
25	161.6 ± 5.1	0.147 ± 0.014	0.18 ± 0.01	25.49 ± 0.78	3.30 ± 0.18	23.75 ± 1.32
50	168.5 ± 1.2	0.218 ± 0.017	0.52 ± 0.03	44.94 ± 2.23	4.50 ± 0.03	38.92 ± 0.29
75	159.2 ± 2.5	0.204 ± 0.010	0.65 ± 0.02	31.8 ± 0.90	4.28 ± 0.10	31.58 ± 0.76
100	156.7 ± 4.6	0.173 ± 0.012	0.64 ± 0.01	24.41 ± 0.42	4.32 ± 0.15	32.92 ± 1.53

Data are presented as mean ± SD, *n* = 3.
